# Advancing the application of systems thinking in health: understanding the dynamics of neonatal mortality in Uganda

**DOI:** 10.1186/1478-4505-12-36

**Published:** 2014-08-08

**Authors:** Agnes Semwanga Rwashana, Sarah Nakubulwa, Margaret Nakakeeto-Kijjambu, Taghreed Adam

**Affiliations:** 1 Information Systems Department, College of Computing and Information Sciences, Makerere University, P.O. Box 7062, Kampala, Uganda; 2 Department of Obstetrics and Gynaecology, College of Health Sciences, Makerere University, P.O. Box 7062, Kampala, Uganda; 3 Kampala Children’s Hospital, Neonatal Pediatrician, Kampala, Uganda; 4 Alliance for Health Policy and Systems Research, World Health Organization, Geneva 1211, Switzerland

**Keywords:** Causal loop diagram, Child health, Health systems research, Methods, Neonatal mortality, Systems thinking, Uganda

## Abstract

**Background:**

Of the three million newborns that die each year, Uganda ranks fifth highest in neonatal mortality rates, with 43,000 neonatal deaths each year. Despite child survival and safe motherhood programmes towards reducing child mortality, insufficient attention has been given to this critical first month of life. There is urgent need to innovatively employ alternative solutions that take into account the intricate complexities of neonatal health and the health systems. In this paper, we set out to empirically contribute to understanding the causes of the stagnating neonatal mortality by applying a systems thinking approach to explore the dynamics arising from the neonatal health complexity and non-linearity and its interplay with health systems factors, using Uganda as a case study.

**Methods:**

Literature reviews and interviews were conducted in two divisions of Kampala district with high neonatal mortality rates with mothers at antenatal clinics and at home, village health workers, community leaders, healthcare decision and policy makers, and frontline health workers from both public and private health facilities. Data analysis and brainstorming sessions were used to develop causal loop diagrams (CLDs) depicting the causes of neonatal mortality, which were validated by local and international stakeholders.

**Results:**

We developed two CLDs for demand and supply side issues, depicting the range of factors associated with neonatal mortality such as maternal health, level of awareness of maternal and newborn health, and availability and quality of health services, among others. Further, the reinforcing and balancing feedback loops that resulted from this complexity were also examined. The potential high leverage points include special gender considerations to ensure that girls receive essential education, thereby increasing maternal literacy rates, improved socioeconomic status enabling mothers to keep healthy and utilise health services, improved supervision, and internal audits at the health facilities as well as addressing the gaps in resources (human, logistics, and drugs).

**Conclusions:**

Synthesis of theoretical concepts through CLDs facilitated our understanding and interpretation of the interactions and feedback loops that contributed to the stagnant neonatal mortality rates in Uganda, which is the first step towards discussing and exploring the potential strategies and their likely impact.

## Background

**Figure 1:**
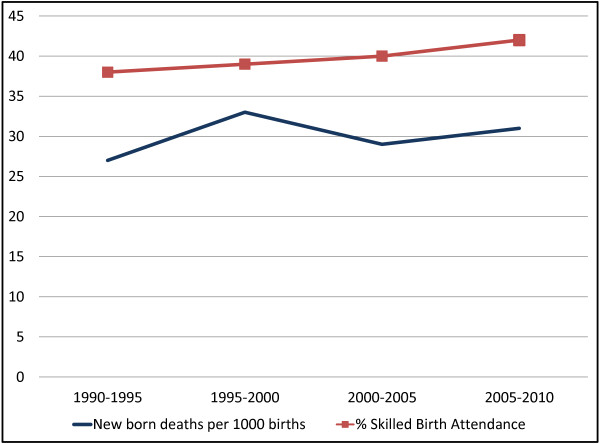
Trends in key neonatal health indicators in Uganda (1990–2010).

With around three million babies dying each year within their first four weeks of life (neonatal period), virtually all (99%) occur in low- and middle-income countries (LMICs) [1]. Moreover, the most recent progress reports on global trends in neonatal mortality have shown alarmingly slow progress, if any, in curbing mortality rates among neonates, the slowest being in sub-Saharan Africa [1]. Three quarters of these neonatal deaths occur within the first week of life and at least 1 million die on the first day of life [1]. Uganda is one of the high burden countries in sub-Saharan Africa where the rate of decline in neonatal mortality has remained below the global average over the past 20 years, with an estimated 28 newborn deaths per 1,000 live births (a total of 43,000 deaths per year) in 2011 [1]. While child survival programs have tended to focus on pneumonia, diarrhoea, malaria, and vaccine-preventable diseases, all of these contributed to death after the first month of life. There is no documented progress in targeted approaches to prevent death around birth and the first week of life (Figure 1).

Several studies have tried to understand the reasons for this slow progress, employing a range of methods including logistic regression models [2-4], cross tabulations [5], principal component analysis, and simultaneous multiple regression models [6]. The vast majority of these studies focused on only one or a few aspects of the problem in isolation. Most notable were lifesaving interventions or key strategies geared towards improving access to, or coverage of, health services. Interventions that do not employ the holistic approach often focus on the symptoms neglecting the underlying root causes, thereby resulting in the reoccurrence of the problem. However, neonatal mortality is a very complex problem involving multiple factors and actors and requiring multiple interrelated and simultaneous strategies to be able to effectively address it, including the difficult challenge of changing attitudes, perceptions, behaviour, and practices [7]. This complexity calls for employing more holistic approaches that acknowledge the complexity of neonatal health and of the health system itself, within which actions need to be taken, monitored, and managed.

Systems thinking provides a means to understand and work with this complexity. It aims to gain insights into the whole by understanding the linkages, interactions, feedbacks, and processes between the elements that comprise the whole system. In many cases, complexity stems from a combination of the complexity of the disease or condition itself (such as neonatal mortality) and the systems in which they are interacting and evolving, in this case the health system [8]. Health systems share the characteristics of complex adaptive systems [9]. They are constantly changing, tightly linked and governed by feedback, hence constantly coping and adapting to actions or changes in other parts of the system. They are history dependent and therefore often resistant to change and new directions, especially those initiated by the stewards of the system [10,11]. Therefore, intervening in the system almost always has ripple effects that affect other parts of the system and introducing change is often not as straightforward as the policy plans and design imply [11,12]. These are just a few of the reasons that argue for using a holistic systems thinking approach that takes into account this intricate complexity.

This study aimed to contribute to this timely debate by exploring: i) how systems thinking tools, more specifically causal loop diagrams (CLDs) [13,14] and system dynamics modelling [15-17], can help better understand the complexity underlying the factors influencing neonatal mortality, particularly in LMICs; and ii) what strategies and leverages may be successful in accelerating progress, using Uganda as a case study. The overall goal was to offer a comprehensive approach to examine the questions that can be applied in Uganda and can be adapted to other conditions, countries, and contexts.

## Methods

**Figure 2:**
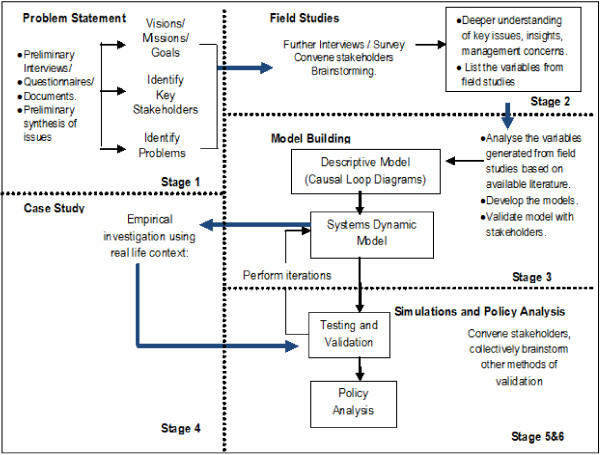
**Research design: dynamic synthesis methodology.** [Williams (2000) [21] revised by Rwashana and Williams (2009) [13].

This study employed the dynamic synthesis methodology (DSM). DSM combines two powerful research strategies, namely the qualitative (case study research method) [18-20] and the quantitative techniques (simulation models) [15-17], to provide solutions to problems. Figure 2 presents the DSM by Williams (2000) [21], later revised by Rwashana and Williams (2009) [13]. DSM has six stages, namely i) problem statement and preliminary data collection; ii) field studies; iii) model building; iv) case study and empirical exploration; v) simulation; and vi) policy analysis. This study applied the first three stages concluding with the development of a refined and validated CLD. The remaining three phases are underway and will be published subsequently.

### Stage 1: problem statement

Preliminary information related to neonatal mortality and the associated problems was collected from peer-reviewed literature as well as global and local reports and policy documents in order to understand and better characterize the current problems influencing neonatal health. The collected data included historical trends of mortality rates and coverage of key related interventions, as well as information describing quality of care, perceptions, views, and attitudes of households and health workers in Uganda.

### Stage 2: field studies

Semi-structured interviews were conducted with various stakeholders including mothers, village health workers, community leaders, front-line health workers at the first-level primary facilities and hospitals, and district and national policy and decision makers (see Additional file 1 for the data collection instruments). The purpose of the interviews was to explore the experiences, views, sources of information, and attitudes with respect to what happens during pre-conception, antenatal care (ANC), delivery and postnatal care (PNC). It also elicited insights on quality and management of health services for women seeking to be future mothers, pregnant women, and neonates. Interview guides were tailored to each type of respondent to cover the relevant range of questions. They were informed by the information gathered during the first stage and brainstorming among the study team of other factors or problems that should be explored. They also allowed for exploring additional aspects or problems raised by the respondents through open ended questions.

### Study population and sampling criteria

The interviews were conducted in Rubaga and Kawempe divisions of the district of Kampala, Uganda, where 47% of the population of Kampala reside. The Rubaga division was purposely chosen because it has two major mission hospitals that provide care for the low- and middle-income population from Kampala and nearby surrounding rural areas. Furthermore, Rubaga has the highest neonatal mortality rate of 54 per 1,000 live births in Uganda, compared to the national average of 27 per 1,000 [22]. The Kawempe division was chosen because it has the only national referral Hospital (Mulago) receiving referrals from all over the country. The sampling frame and selection criteria for the different interviews are presented in Table 1.

**Table 1:** Sampling frame and selection

**Category**	**Sample size**	**Sampling approach**	**Criteria**	**Comment**
Mothers	282	Random sampling	Mothers who delivered in the last 12 months residing in Rubaga and Kawempe divisions	Overall, 274 interviews were considered; 8 had significant missing information
Village health workers (VHWs) and community leaders	16 VHWs and 10 community leaders	Convenience sampling approach	VHWs and community leaders residing in Rubaga and Kawempe divisions	We stopped identifying new interviewees when no new issues were raised in the last interviews
Frontline health workers (FHWs)	20 FHWs (13 nurse/midwife/clinical officers and 7 doctors)	Purposive sampling inclusive of both government and private health units in the two divisions.	Officers-in-charge of the facility and/or health workers providing voluntary counselling and testing, or prevention of mother to child transmission services	One staff was interviewed from each available cadre/level
Healthcare decision and policy makers	7 leaders/policy makers	A purposive sampling approach.	Selected on the basis of their role in formulation and implementation of neonatal health policies	All the leaders/policy makers were interviewed

#### ***Mothers***

A random sampling approach was used to identify mothers in antenatal clinics and from homes in the villages of Rubaga and Kawempe. At 95% confidence interval, a neonatal death rate (p) of 5.4%, and a level of permissible error (e) of ≤10%, the sample size was determined (see Additional file 2 for sample size calculation). The sample size was estimated as 282 mothers, divided equally between Rubaga and Kawempe (i.e., 141 each). In each division, 85 of the 141 mothers were sampled from health facilities and 56 from home. Mothers who delivered in the last 12 months and had consented to the study were included in the study, regardless of whether their babies were alive or dead. Mothers whose last child was more than 12 months old or was mentally ill were not included. Although the planned interviews were 282, only 274 interviews (Kawempe: 51 home, 88 health facilities; Rubaga: 55 home, 80 health facilities) were considered in the analysis. The remaining 8 interviews had significant missing information.

#### ***Village health workers and community leaders***

A convenience sampling approach was used to select the respondents by selecting those residing in the villages where interviews with mothers and health workers occurred. A total of 16 village health workers and 10 community leaders from the villages of Rubaga and Kawempe were interviewed. We stopped identifying new interviewees when no new issues were raised in the last interviews.

#### ***Front-line health workers***

Twenty front-line health workers were selected from Kawempe and Rubaga health facilities. The health facilities were purposively selected as follows:

– Kawempe: Mulago National Referral Hospital, one private hospital, Kawempe government health centre IV and three private health centres.

– Rubaga: Mengo hospital (mission hospital private not-for-profit), one private hospital, two private health Centres, and two government health centre III.

During the interviews with the in-charge of the facilities, health workers providing voluntary counselling and testing or prevention of mother to child transmission services were identified. Among the health workers who are performing these services, one staff was interviewed from each available cadre/level, purposively selected in discussion with the in-charge. The total sample included 13 nurse/midwife/clinical officers and 7 doctors from public and private health centres and hospitals in both divisions.

#### ***Healthcare decision and policy makers***

A purposive sampling approach was used to select 7 leaders/policy makers, on the basis of their role in formulation and implementation of neonatal health policies. These include two heads of the obstetrics and gynaecology departments of hospitals in each of the selected divisions; two neonatologists, one in a private and one in a public hospital; one district health officer, the person in charge of women and children issues at the district headquarters; one division health officer; and one ministry official involved in neonatal health.

### Ethical consideration

Ethical approval was obtained from Mengo Hospital Research Review Committee and the National Council of Science and Technology of Uganda. Consent forms were prepared to protect and ensure the dignity and welfare of all participants, as well as those who may be affected by the results of the research project. All participants were asked to sign a consent form and were informed that participation was voluntary and that they could opt out at any moment. Anonymity was ensured by using study identification numbers and initials rather than names of individuals.

Mothers who were able to read and write filled in the questionnaire, while those who could not were interviewed. The socio-demographic characteristics of the mothers who interviewed for those are presented in Table 2. The age distribution, household income, and number of mothers who lost at least one neonate was similar to national average, while mothers’ education, percentage that were housewives, and the number of pregnancies was closer to the urban rather than national rates [22,23].

**Table 2:** Socio-demographic characteristics of mothers interviewed during the field studies in two divisions of Kampala District, Uganda, n = 274

**Variable**	**Category**	**n (Percentage)**
Age	15–20	49 (17.9)
	21–30	162 (59.1)
	31–40	59 (21.5)
	40+	4 (1.5)
	**Total**	**274 (100)**
Marital status	Married	209 (76.4)
	Not married	64 (23.2)
	Widowed/divorced	1 (0.4)
	**Total**	**274 (100)**
Highest level of education	None	9 (3.3)
	P1–P7	72 (26.2)
	Secondary education	140 (51.7)
	Post-secondary education	51 (18.8)
	**Total**	**274 (100)**
Occupation	Farmer	5 (1.8)
	Housewife/does not work	122 (44.7)
	Health worker	9 (3.3)
	Teacher	13 (4.8)
	Business woman	94 (34.1)
	Other professions	31 (11.4)
	**Total**	**274 (100)**
Household income per month (UGX) (1 USD = 2,500 UGX)	Below 50,000	34 (12.4)
	50,000–100,000	66 (24.1)
	Above 100,000	144 (52.6)
	Not indicated	30 (10.9)
	**Total**	**274 (100)**
Number of pregnancies	1–3	204 (74.4)
	4–6	63 (23.1)
	7+	7 (2.6)
	**Total**	**274 (100)**
Had lost neonate	Yes	18 (6.6)
	No	256 (93.43)
	**Total**	**274 (100)**

### Stage 3: model building and validation of the causal loop diagrams

First, we pooled the different sources of data from stages one and two and categorised these according to the following themes: factors associated with the mothers’ attendance to health services; social/personal characteristics associated with mothers’ attendance to health services; factors that contribute as well destroy the health of neonates; factors associated with the health service delivery in health facilities; and factors in the community and family that are associated with mothers’ attendance to health services.

We then used thematic analysis to compile and analyse the qualitative data. Descriptive statistics and cross tabulations were used to explore the quantitative data. SPSS 10.0 was used for these analyses [24]. Using the findings, we brainstormed and generated a list of potentially important variables that are associated with neonatal mortality, which was used to develop descriptive CLDs using Vensim Software [25]. The full list of variables considered for this analysis are presented in Additional file 3, which includes variables that were not considered in this analysis as well as evidence of association existing in the literature; this was not supported by our empirical findings for this case study.

### Development of casual loop diagrams

**Figure 3:**
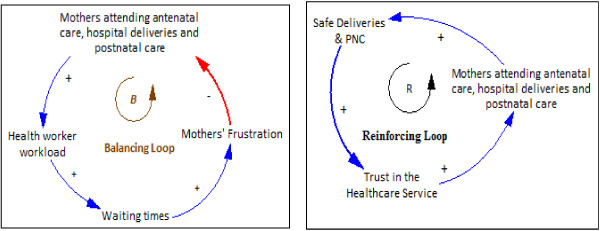
Example of balancing and reinforcing loops.

CLDs help us to understand and depict the feedback mechanisms that are generated within complex systems which include the relationships, dynamics, and delays associated with the variables that generate them. They offer a practical way to understand and express the systems’ interrelated parts and the cause-effect linkages for the problem in question. CLDs are composed of two components; variables and influences (links). An influence has direction shown by an arrow and an indicator as to whether the influenced element is changed in the same (+) or opposite (-) direction as the influencing element. That is, a link from element A to element B () may be positive if a change in A produces a change in the same direction, or negative () if a change in A produces a change in B in the opposite direction. A change in element A which produces a change in element B only after a delay is denoted by.Feedback loops occur when arrows connect a variable to itself through a series of other variables. There are two main types of feedback loops that can be expressed using CLDs: balancing and reinforcing loops, as illustrated in Figure 3. Balancing loops apply where there is an attempt to solve a problem or achieve a goal. They are also called neutralizing loops, where cause and effect cycles seek to counter a change with a push in the opposite direction. Figure 3 shows a balancing loop whose goal is to increase the mothers’ participation in health services. As more mothers participate in health services, the workload increases, thus increasing the waiting times resulting in frustration, which in turn lowers the participation. Reinforcing loops represent a growing action where each action adds to another and may be referred to as virtuous cycles when they produce desirable effects or vicious cycles when they produce negative effects. Figure 3 also shows a reinforcing loop where growing participation in health service arising from safe deliveries results in increased trust, which further increases participation.

### Validation of the causal loop diagrams

Validation of the CLDs and the underlying conceptual thinking was carried out by nine local and international neonatal and maternal health stakeholders, including both researchers and implementers, as illustrated in Table 3.

**Table 3:** Experts involved in the causal loop diagram validation (n = 9)

**Position**	**Affiliation**	**Number**
Head of Paediatrics Department	Mengo Hospital, Uganda	1
Obstetrician and Gynaecologist	Mulago Referral Hospital, Uganda	2
Lecturer, Department of Obstetrics and Gynaecology	College of Health Sciences, Makerere University, Uganda	1
Nursing Officer In-charge of Maternity Department	Mengo Hospital, Uganda	1
Head, Obstetrician and Gynaecologist	Mengo Hospital, Uganda	1
Paediatrician and researcher in maternal, neonatal, and child health issues	Universidad Peruana Cayetano Heredia and Universidad Nacional Mayor de San Marcos, Lima, Perú	1
Paediatrician/Professor	Department of Global Public Health and Primary Care, University of Bergen, Norway	1
Paediatrician/Neonatologist	WHO, Coordinator, of the maternal, neonatal and child research and development team	1

Respondents were asked to state whether all the variables and relationships in the CLDs existed and whether there were any significant causal factors missing. In case there were some factors missing, they were asked to list them. In addition, respondents tested whether the directions of each of the links were right or needed to be reversed (implying that the effect is the cause) and were asked to state whether there were other effects that could be observed as a result of the causes in the CLDs. The validation instrument also included explanations of the objectives of the validation exercise, the meaning of the elements used in the CLDs, and how to respond to the questions; see Additional file 4 for the validation instrument. Suggested modifications from the validation exercise were discussed by the researchers and used to further improve the CLDs together with further qualitative analysis of the collected data in response to questions raised by the validators, leading to the CLDs presented below.

### Findings

Responses from all the interviews were categorized into demand and supply issues. The demand side captures the issues associated with the uptake of health services while the supply side shows the issues associated with the supply of health services. The key findings are presented in turn below.

### Demand side issues

#### ***Pre-conception***

Overall, 44% (8/18) of the mothers who lost their neonates suffered from diseases before pregnancy, including HIV, high blood pressure, malaria, sickle cell, and diabetes, among others.

#### ***Antenatal care***

While 97.8% of the mothers in our sample attended ANC at least once, 25.9% of them attended fewer times than recommended, providing the following reasons for failure to attend: lack of money for transport, busy work schedules, attending school, and delayed start of ANC clinic.

#### ***Delivery***

The majority of the mothers (97.4%) were provided transport to the health centre for deliveries by the community (56.9% spouse; 17.2% relatives; 9.0% friends, and 3.0% neighbours). Only 13.1% used their money for transport.

#### ***Postnatal care***

Some of the respondents lacked knowledge on how to care for the babies. For example respondents stated that they used vaseline, herbs, and powder for cord care. Some used breast milk, herbs, urine, water, and saline to care for swollen eyes.

#### ***Attitudes and beliefs***

While almost all mothers (97.4%) in our sample gave birth at a health facility and believed in the importance of doing so, 40.9% stated that they knew of mothers in their community who did not go to health facilities for their deliveries. Cited reasons for why some of these mothers may have chosen not to go to health facility for their deliveries included traditional beliefs (14.1%), religious beliefs (3.2%), lack of permission from the spouse (3.8%), and lack of trust in the health system (14.7%). On the latter, more elaborations from the mothers included that they knew of mothers who feared that health facilities were poorly equipped, had insufficient health workers, were overcrowded with long waiting lines, lacked 24-hour care, had careless, rude, and abusive health workers who carried out excessive episiotomy, and that they found seeking care at health facilities to be very costly, possibly due to unavailability of drugs and lack of free supplies at health centres such as the Free Mama Kits for those who do not have their own.

#### ***Sources of information***

Mothers stated that they generally obtain health information from various sources including radio, friends, brochures, films, health workers, family, and newspapers. With respect to information provided to the mothers during ANC, 92.8% of the mothers received information on HIV and the value of HIV testing, 84.6% on family planning, 84.3% on breast feeding, and 83.2% on nutrition (83.2%). When asked about what they thought were the best methods to encourage mothers to attend ANCs and give birth at health facilities, they listed the following: home visits by health workers, village meetings, social meetings, community notices, and health education during ANC sessions.

### Supply side issues

#### ***Quality of health service and hygiene***

Although several mothers perceived hygiene at the health facilities to be generally good, 71% of the mothers reported that hygiene was still in need of further improvement. The surveyors also observed that some of the facilities were not well maintained and infection prevention was not well observed. Community leaders also noted that there were cases of neglect by health workers; one leader said “*… babies born with … no one to wrap them*”.

#### ***Health workers motivation***

All of the interviewed health workers mentioned that they were poorly paid. It was also observed by the mothers and the surveyors that staff experienced burnout due to workload arising from few skilled workers at the units resulting in a high provider to number of deliveries ratio. Health workers stated that both remuneration and safety measures at the health facility would increase their motivation. They also stated other demotivating problems such as electric power supply breakdowns during delivery, mothers losing a lot of blood before or after delivery especially where no blood bags were available, and difficulty in getting mothers to a referral hospital in case of emergency due to lack of ambulances.

#### ***Availability of supplies and equipment***

Staff voiced frustration due to unavailability of the necessary equipment and supplies. According to our survey, 34% of health workers had deficiencies in resuscitation equipment and 67% lacked ultrasound facilities.

#### ***Record keeping***

Only 16.8% of the health workers in our sample prepared births and death certificates and 83% kept Health Management Information System child health records and submitted them to higher levels; 50% of the village health workers reported that they lacked birth registers.

#### ***Policy enforcement***

Community leaders and policy makers noted that some policies and guidelines were not readily available to the public and were not always adhered to. Only 33% of the health facilities had clinical guidelines available to them. For example, guidelines on cord care were not readily available. It was also noted by the policy maker at the district level that there was no clear policy and enforcement on recruitment of appropriate numbers of front-line health workers for the population.

#### ***Supervision***

Overall, 18.8% of the village health workers reported that they were not supervised, which is consistent with community leaders perceptions of gaps in supervision at the community level. Similarly, supervision of health workers at health units was also considered poorly enforced. In addition, community leaders voiced concerns about the number of unqualified people treating the population in their communities and that efforts to control this health risk should be strengthened.

### Causal loop diagrams

**Figure 4:**
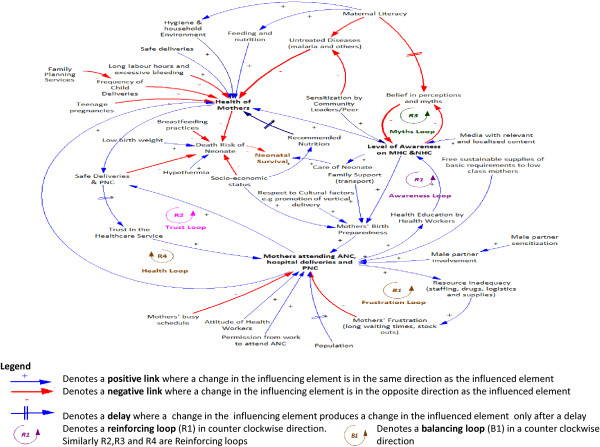
Causal loop diagrams showing the demand for neonatal and maternal health service.

**Figure 5:**
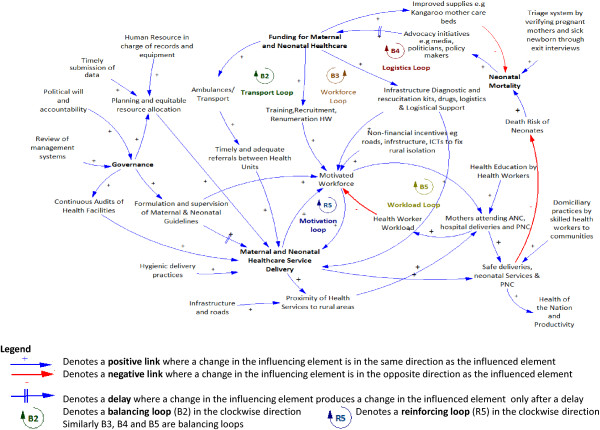
Causal loop diagram showing the supply of neonatal and maternal health service delivery.

Two CLDs depicting the factors associated with the demand for (Figure 4), and supply of (Figure 5) health services for neonates and mothers were created from the interview and the data collection in stage one, together with brainstorming among the study authors. Several reinforcing and balancing feedback loops can be observed in these CLDs. A detailed analysis of the CLDs is provided below:

#### ***Dynamics of the demand for neonatal and maternal healthcare service***

The dynamics involved in the demand for neonatal and maternal healthcare are presented in Figure 4. We identified one balancing loop (B1, frustration loop) where there is an attempt to achieve the goal of increasing uptake of maternal health service and four reinforcing loops (R1, awareness loop; R2, trust loop; R3, myths loop; and R4, health loop) that represent growing actions as illustrated below.

The awareness loop (R1) is a virtuous cycle that enhances the growth of awareness. The level of awareness of neonatal and maternal health issues results in improved health of mothers and increased attendance to neonatal and maternal healthcare services, thereby lowering the death risk of neonates. Awareness is enhanced through health education programmes provided during ANC visits, media (TV, radio, newspapers), sensitization by community leaders, and word of mouth through peer to peer interactions among mothers. As mothers attend ANC, PNC, and hospital deliveries, the level of awareness increases resulting in mothers’ preparedness for birth. Mothers’ birth preparedness, which is achieved with increased level of awareness, family and community support, and socio-economic status increases the likelihood of attending health services and therefore having safer deliveries, and further enhances the attendance to ANC. The growth in the awareness loop eventually slows down due to the inadequacy of resources exhibited in the frustration loop resulting into the limits to growth archetype (loops R1 and B1). In order to avoid the limits to growth, the quality of service in the health facilities must be sustained.

The trust loop (R2) enhances the trust of women in health systems through provision of safe health care deliveries and PNC. As more mothers attend ANC, hospital deliveries, and PNC, the level of safe deliveries and PNC increases, which in turn increases their trust in the healthcare service. The growth in the trust loop eventually encounters limiting action thereby exhibiting the limits to growth archetype (loops R2 and B1). The limits to growth of this cycle arises from inadequate resources that are needed to sustain the quality of maternal and neonatal healthcare service deliveries as exhibited in the frustration loop (B1) explained below. In order to maintain the trust, the quality of the maternal and neonatal service must be observed.

The frustration loop (B1) shows that the desired state is to have as many mothers attending ANC, healthcare deliveries, and PNC. Attendance to ANC and delivering at health facilities plays a big role in promoting safe deliveries and obtaining PNC, which will also contribute to increased trust in the health system and improving the general awareness about the benefits of these health services in the community. When the number of women participating in maternal and neonatal health services increases, the resources (staff, drugs, logistics, and supplies) in the health facilities are depleted, leading to frustration resulting from effects of poor service delivery such as long waiting times and drug stock outs, which results in a decrease in attendance, thus demonstrating a balancing loop. Efforts should be made to ensure that the resources in the health facilities match the demand, thereby minimising frustration of mothers.

The myths loop (R3) produces a desirable effect whereby beliefs in myths are decreasing. As the level of awareness on maternal health care (MHC) and neonatal health care (NHC) increases, belief in perception and myths decreases. As the belief in perceptions and myths decreases, the level of awareness increases. Belief in myths and perceptions that are enhanced as a result of low maternal literacy levels are a hindrance to the level of awareness. Efforts to keep the growth of awareness through community and peer to peer sensitization, health education, and media should be made so that eventually the myths die off.

The healthy mothers loop (R4) produces a virtuous cycle where mothers’ attendance to ANC and hospital deliveries results in improved mothers’ health, thereby producing safe deliveries, which builds trust resulting in a further increase in the mothers’ uptaking of health services. This loop interacts with the frustration loop creating the limits to growth archetype.

The CLD shows that neonatal health heavily depends on the health of the mothers. The health of the mothers can be increased by increased self and household hygiene, increased level of awareness, attendance to ANC, PNC, and health facility deliveries, and adherence to the recommended nutrition. Factors that lower the mothers’ health include increased frequency of child delivery, diseases such as malaria, and teenage pregnancies, among others. The death risk of a neonate increases with hypothermia, poor breast feeding practices, poor socioeconomic status, and poor care of the neonate resulting from lack of awareness.

#### ***Dynamics of the supply for neonatal and maternal healthcare service***

The dynamics involved in the supply of neonatal and maternal health services are presented in Figure 5. In this CLD, one reinforcing loop (R5, motivation loop) that represents a growing action in the motivation of the workforce and four balancing loops (B2, transport loop; B3, workforce loop; B4, logistics loop; and B5, workload loop) all representing desired goals towards improvement in the supply of maternal health service are identified and explained below.

The transport loop (B2) emphasizes the importance of having timely and adequate referrals between hospitals towards improvement of the maternal and neonatal healthcare services, which can be achieved through the provision of transport/ambulances. As the funding for maternal and neonatal healthcare increases, provision of ambulances and transport increases, resulting in timely and adequate referrals between health units. This improves maternal and neonatal healthcare service delivery which lowers the death risk of neonates and in turn lowers neonatal mortality rates. A rise in neonatal mortality rates attracts an increase in advocacy initiatives resulting in increased funding. Funding for purchase and maintenance of vehicles as well as policies for use of these vehicles should be done.

The workforce loop (B3) shows that the desired state is to have a motivated workforce in terms of having the right skills, remuneration, and attitude. An increase in the funding for maternal and neonatal healthcare results in an increase in the training, recruitment, and remuneration of health workers, resulting in a motivated workforce. This improves maternal and neonatal healthcare service delivery, which lowers the death risk of neonates and in turn lowers neonatal mortality rates. A rise in neonatal mortality rates attracts an increase in advocacy initiatives resulting in increased funding. For this to happen, governments must be willing to fund and invest in the training, recruitment, and remuneration of health workers. Failure to do so eventually results in poor maternal and neonatal healthcare, thereby increasing the death risks of neonates and resulting in increased neonatal mortality.

The logistics loop (B4) shows that the desired state is to have quality maternal and neonatal health services where the health facilities have logistics, drugs, and resuscitation kits. As the funding for maternal and neonatal healthcare increases, provision of infrastructure, diagnostic and resuscitation kits, drugs, and logistics and logistical support increase. This improves maternal and neonatal healthcare service delivery, which lowers the death risk of neonates and in turn lowers neonatal mortality rates. A rise in neonatal mortality rates attracts an increase in advocacy initiatives resulting in increased funding. Governments should provide adequate funding to ensure that the required resources are made available.

The motivation loop (R5), together with the workload loop (B5), make up a limits to growth archetype. Limits to growth of this loop result from exceeding the capacity of mothers that can be handled by the workforce. The motivation loop shows that a motivated workforce that is well supervised, remunerated, and trained, and with adequate supplies will increase the maternal and neonatal healthcare service delivery. When the service delivery is good, this, in turn, further motivates the staff resulting in a virtuous cycle. The workload loop (B5), on the other hand, shows that an increase in a motivated workforce increases the number of mothers attending ANC, hospital deliveries, and PNC, which in turn increases the workload thus lowering the workforce that is motivated. It is therefore important that the health services and workforce are upgraded to meet the growing population which will even out the number of patients attending the few health facilities.

With adequate financing of maternal and neonatal health services, the following can be made available: training, recruitment, and adequate remuneration of health workers, resulting in a motivated workforce providing quality healthcare service and thus leading to safe deliveries; transport for timely and adequate referrals between health units and obtaining blood bags if necessary; and equipment (e.g., for resuscitation, suction or oxygen), medical supplies, emergency drugs, and safe delivery kits. The availability of funding coupled with good governance is necessary for the formulation and enforcement of healthcare guidelines, planning, supervision, and efficient and equitable resource allocation, as well as monitoring and evaluation and audits of health facilities.Although the graph in Figure 1 demonstrates that the skilled birth attendance has increased slightly there has not been significant decline in neonatal mortality rates over the past two decades. This clearly brings out the various limits to growth loops identified in the CLDs, which show that as the limits to growth are reached, the growth engine loses its effectiveness, and the growth curve begins to flatten.

### Validation of the causal loop diagrams

Respondents used the validation instrument in Additional file 4 to test the CLDs. The validation process contributed to further modification and led to the final CLDs presented in this paper. The respondents were also asked to generally assess their experience with viewing the issues related to neonatal health by means of the CLDs. All the respondents saw and understood the CLD for the first time. The objective was to determine whether they found the CLDs to be reasonable, representative of the healthcare issues, and whether they were useful aid and communication tools. See Table 4 for their perceptions.

**Table 4:** Overall impressions of the experts regarding the CLDs

**Parameter**	**Rating categories**	**Number of respondents**
Were they reasonable (realistic)?	Very reasonable	2
	Reasonable	6
	Fairly reasonable	1
	Not reasonable	
How well did they represent issues related to neonatal health services?	Very good	4
	Good	5
	Fairly good	
	Not at all good	
Are they useful as a communication tool?	Very useful	2
	Useful	6
	Fairly useful	1
	Not at all useful	
Are they a useful aid tool that can be used by stakeholders in decision making?	Very useful	3
	Useful	5
	Fairly useful	1
	Not at all useful	

## Discussion

This study presents the first of its kind in-depth analysis of the possible causes of neonatal mortality in a given context with an explicit focus on complexity. We explicitly examined the feedback loops that were generated due to the complexities surrounding neonatal mortality as a first step towards considering and testing alternative short- and long-term strategies that may be used to efficiently address the root causes of some of these problems.

One of the main points of strength in this study is the inclusion of a wide range of perspectives of the different key stakeholders, including the mothers, front-line health workers, and village health workers. This undoubtedly enriched the analysis and provided a deeper understanding of the real causes of neonatal mortality and its interplay with the complexities of the health system it interacts with [9]. The second strength of this paper is using a validation instrument to validate the CLDs, which strengthened our model and its global relevance, given that we also approached international stakeholders from various regions of the world.

A limitation of this study is that it was undertaken in Kampala district. It is possible that there are other factors that are peculiar to other parts of Uganda such as the geographical environment (terrain), extreme poverty, and rural levels that have not been captured in this research. Our findings may, therefore, be more representative of urban Uganda than the whole country. However, this is only relevant for quantitative studies. The main objective of our study was to exploit the strength of qualitative approaches to explore how this problem, and its intricate complexities, can be understood in-depth, using a systems thinking approach. The objective of our study, therefore, included a methodological component and is not only relevant to neonatal health but to other diseases and settings.

The findings from the field studies suggest several short- and long-term strategies that would bear fruit in reducing the burden of neonatal mortality. For example, as shown in other studies [26], 44% (8/18) of the mothers who lost their neonates suffered from diseases before pregnancy. Ensuring that women in the age-bearing period receive adequate health education on their own health and its contribution to child outcomes prior to conception could, therefore, significantly increase the likelihood of detecting and addressing some of these problems, especially since several are preventable or treatable. The same applies for health education of postnatal health problems to avoid some of the harmful practices, such as those observed for cord care in our sample. Health education is also likely to increase coverage of ANC and deliveries at health facilities as reported by Uddin and Hossain [27] and Midhet and Becker [28]. For example, 97% of the mothers in our study visited ANC at least once and more than 80% of them had good knowledge about the main health problems that are relevant to pregnancy and labour.

Our findings also highlighted the importance of supportive spouses and community involvement and its contribution to higher utilization of health services, especially for delivery, where the majority of the women (97.4%) received support by the community and only 13.1% used their money for transport. Our findings also highlighted health system problems that are standing against any possible improvement in neonatal mortality. These include the situation of health facilities in terms of hygiene and infection control, lack of cheap life-saving equipment and supplies, such as resuscitation kits, and suction machines.

### Leverages

Leverages are influences within the system where small changes can effect a substantial change in the system. From the analysis of the CLDs, the following were perceived as high leverage points which can effect significant improvement in neonatal healthcare:

• Increased awareness on maternal and neonatal healthcare can weaken the vicious cycle exhibited by the myths loop (R3) while strengthening the virtuous cycle of the awareness loop (R1). Mothers’ awareness on the recommended feeding, nutrition, hygiene, household environment, and mothers’ birth preparedness and efforts to avoid untreated diseases results in improved health of the mothers, which in turn lowers neonatal mortality rates. Some of the short-term interventions which may improve awareness include aggressive advertising, campaigns, sensitization, and education of the women and girl child as well as increasing the effectiveness of the health education sessions during ANC and PNC. Special gender considerations to ensure that girls receive essential education thereby increasing maternal mortality rates is a longer term strategy but would synergistically address many other health and non-health issues.

• The low socio-economic status is a key determinant in the health of the mothers and the neonates. With improved socio-economic status, mothers are able to obtain the recommended nutrition, healthcare, and the requirements for birth preparedness. While introducing incentives, such as transport vouchers and free birth kits for pregnant women, would motivate them to attend ANCs and enable them to be better prepared for health facility deliveries in the short term, the government should work towards improving the socioeconomic status of the nation.

• Funding for maternal and neonatal health care should be prioritized at the national level. Efforts by the government and policy makers to upgrade the health service infrastructure as well as build systems for monitoring the resources (staffing, drugs, and stocks) would go a long way in minimizing the effects arising out of the frustration loop B1. Improved maternal and neonatal health service delivery will strengthen the virtuous cycle created by the motivation loop R5. In addition, without a motivated health work force that is well trained, adequately remunerated, and with an acceptable workload there is not much to be expected in terms of the quality of the care provided nor the likelihood that mothers will come to seek care at health facilities. Other short- and long-term strategies may include improved supervision and internal audits at health facilities to ensure that maternal and neonatal guidelines are adhered to as well as establish the current conditions and gaps in resources (human, logistics, and drugs) to guide the funding for national health care.

This study reports on stages 1–3 of our research design. Future work involves the completion of stages 4–6, where we will use empirical data to develop the quantitative (simulation) model including testing of different policy options. Iterations to test and validate the model will be conducted through brainstorming sessions with stakeholders. What-if analysis will be used to test different strategies that have been suggested by this research and by stakeholders, including policy makers, during brainstorming and validation workshops with the aim of improving policy analysis and design in neonatal health. The model will be used to determine the strategies that could have a great impact on neonatal mortality using sensitivity analysis.

## Conclusions

This study adopted a systems thinking approach to capture and analyse the interactions between technical, policy, behavioural, and cultural issues related to neonatal mortality. It provides a broad integrated view of the dynamics associated with neonatal health, thus accommodating the different stakeholder viewpoints. The synthesis of the various theoretical concepts through the use of CLDs facilitated the understanding and interpretation of the different interacting elements and feedback loops that contributed to the stagnant neonatal mortality rates in Uganda, which is the first step towards discussing and exploring the pros and cons of the different strategies and the priorities that should be addressed based on their likely impact and cost-effectiveness.

This paper also illustrated the importance of validation of the structure and relationships of the CLD with key stakeholders, including decision makers, which was beneficial, enriching, and ensured that the variables of the CLDs represent that of the real system. The validation exercise demonstrated that CLDs can help the different stakeholders view complex health problems from different perspectives and facilitate shared understanding and common ownership of the interpretations of health problems. They also provide a broad integrated view of the problems which can be used for learning and process improvement, as well as operational management. The methods, approaches, and findings from this study are not only applicable to neonatal health and Uganda, but also to other settings and questions of a similar nature.

## Abbreviations

ANC: Antenatal care; CLDs: Causal loop diagrams; DSM: Dynamic synthesis methodology; LMICs: Low- and middle-income countries; PNC: Postnatal care.

## Competing interests

The authors declare that they have no competing interests.

## Authors’ contributions

ARS conceived the paper, developed the study design and data collection tools, analysed the data, developed the CLDs, and drafted the manuscript. SN and MNK contributed to the study design and data collection and analysis and the interpretation of the results. TA contributed to formulating the study design, data collection, interpretation of the results, and of drafting the manuscript. All authors reviews and approved the final version.

## Supplementary Material

Additional file 1 Data collection instrument. Click here for file

Additional file 2 Sample size calculation. Click here for file

Additional file 3 List of variables. Click here for file

Additional file 4 Causal loop diagram validation instrument. Click here for file
