# Supplementary material for: Advancing the application of systems thinking in health: understanding the dynamics of neonatal mortality in Uganda
Source: Health Res Policy Syst. 2014 Aug 8;12:36. doi: 10.1186/1478-4505-12-36 (PMC4134459; doi:10.1186/1478-4505-12-36)
Supplement: Additional file 1 — Data collection instrument. [file 1478-4505-12-36-S1.docx]

# Additional file 1 – Data collection instruments

**Consent form**

**Form Number [** ]

| Confidential, information to be used for research purposes only |  |
| --- | --- |
| **Study Title : A Systems Thinking Approach to Understanding Neonatal Mortality**  **MOTHERS’ ASSESSMENT INSTRUMENT FOR NEONATAL PRACTICES** | |

I am _____________________________a researcher, participating in the study titled “A Systems Thinking Approach to Understanding Neonatal Mortality”. The aim of the study is to investigate factors associated with neonatal mortality rates in order to facilitate deeper understanding of the problems and to generate insights that could possibly lower the neonatal mortality rates. We are conducting a survey about the health of mothers and infants to help us establish the problems associated with the health of infants in particular the Newborn. Therefore, I would like to interview a mother with a young infant in this household to get the above information. You are free to respond or not but we hope you will respond, the information is highly confidential and will be used to generate insights towards improvement of neonatal healthcare.

**Screening question**

*Is there any woman living in this house who delivered in the last 12 months? 1. Yes 2. No **(*If “YES” fill in questionnaire)***

**PARTICIPANT CONSENT**

I ____________________________________________ **[Name of participant (print)]** have read or have had read to me the above document for the study titled “A Systems Thinking Approach to Understanding Neonatal Mortality ” and that it has been explained to me and I understand it. I have been given an opportunity to have my question\s about the study answered to my satisfaction. I agree to participate voluntarily.

__________________ _________________________________________

Date Signature or mark of participant

_________________________________________

Name of participant (print)

**If participant cannot read the form herself, a witness must sign here:**

I was present while the informed consent form was read to ______________________________________ **[Name of participant (print)]**. All questions by the participant were answered and the participant has agreed to take part in the study.

__________________ _____________________________________

Date Signature of witness

_____________________________________

Name of witness (print)

| **MOTHERS’ ASSESSMENT INSTRUMENT FOR NEONATAL PRACTICES** |
| --- |

This is a study titled ”A Systems Thinking Approach to Understanding Neonatal Mortality” with the aim of investigating the factors associated with neonatal mortality rates. This survey is about the health of mothers and infants and is designed to help us establish the problems associated with the health of infants in particular the Newborn.

| **Section 1: General Information** | | |  |
| --- | --- | --- | --- |
| 1 | Sub County : |  |  |
| 2 | County : |  |  |
| 3 | Place of interview | 1. Home  2. Health Centre III  3. Health Centre IV  4. Private Health Centre  5. Private Hospital  6. Referral Hospital  7. Other (specify)………………………… |  |
| 4 | Age | 1. 15 – 20  2. 21– 30  3. 31– 40  4. above 40 years |  |
| 5 | What is the highest level of school you attended? | 1. None 2. P1 – P7 3. Secondary 4. Post Secondary |  |
| 6 | Are you currently married living with a man? | 1. Yes 2. No |  |
| 7 | If yes, what is the occupation of your spouse? | 1. farmer 2. housewife (stays at home) 3. health worker 4. business woman 5. teacher 6. Not working 7. other (specify) …………………… |  |
| 8 | What is your occupation - what kind of work do you mainly do? | 1. farmer 2. housewife (stays at home) 3. health worker 4. business woman 5. teacher 6. Not working 7. other (specify) ………………… |  |
| 9 | What is your estimated household income per month (Uganda Shillings)? | 1. below 50,000/= 2. 50,000 -100,000/= 3. above 100,000/= |  |
| 10 | How many pregnancies have you had ? | 1. 1-3 2. 4-6 3. 7 and more |  |
| 11 | How many of your children are alive ? | 1. all (skip to question 19) 2. none   3.other 3. specify ………….. |  |
| 12 | For those babies who passed away, did any of the children die before they were 28 days old (1 month old) | 1. Yes 2. No (skip to question 19) |  |
| 13 | How old was the baby when he/she died? | At birth ……………………...1  1 – 6 days…………………….2  7 – 28 days……...................3 |  |
| 14 | What was the weight of the baby? | Less than 2.5 Kg………………1  2.5Kg – 5 Kg…………………….2  Over 5Kg………………………….3  I do not know ………………….4 |  |
| 15 | If Yes, what was the cause of death? | 1. Due to mother’s labour complications 2. Neonatal infections 3. Pre-term and low birth weight 4. Hypothermia 5. Congenital malformation 6. Abuse by the husband 7. Others (specify) ………………… |  |
| 16 | Was the baby delivered dead or not? | Fresh still birth ……………..1  Macerated …………………. 2 |  |
| 17 | What was the sex of child | Male……………………………….1  Female……………………………..2 |  |
| 18 | What was the order of pregnancy | …………………………..number |  |

| **Section 2: Pre-conception** | | |  |
| --- | --- | --- | --- |
| 19 | Were you using any family planning method prior to getting your last pregnancy? | 1. Yes 2. No |  |
| 20 | If yes, what method of family planning were you using | 1. Pills 2. Injection 3. Foaming tablets 4. Condom 5. Female sterilization 6. Periodical abstinence (rhythm) 7. Withdrawal 8. Abstinence 9. IUD (Coil) 10. Others |  |
| 21 | Before you started giving birth, did you get any counseling about nutrition / diet in reference to preparation for pregnancy? | 1. Yes 2. No |  |
| 22 | If “**YES**’, what were you told about nutrition / diet? ***(Explain)***  ***(e.g. type of food, why that food etc)*** |  |  |
| 23 | Who talked to your or counseled on nutrition or diet? |  |  |
| 24 | Before pregnancy, did you suffer from any medical condition? | High Blood Pressure …....………1  Diabetes ………………..…………… 2  Sickle Cell Anaemia………. ……..3  HIV…….. ……………………..…..… 4  Others (specify)…………….……. 5 |  |

| **Section 3: Antenatal Care** | | | | | | |
| --- | --- | --- | --- | --- | --- | --- |
| **Q. #** | **Question** | | **Codes** | | |  |
| 25 | Did you attend antenatal care during your last pregnancy ? | | Yes………………………………1  No……………………………….2 | | |  |
| 26 | If yes, did you have an antenatal card for your pregnancy?  ***(Check the card if available)*** | | Yes………………………………….…..….1  Lost it……...………………………...…..2  Is kept in the institution………….3  Never had one……………………..…4 | | |  |
| 27 | If you attended ANC, where did you receive antenatal care**?**  **(MULTIPLE RESPONSE)** | | Private Hospital…………………………1  Gov. Hospital…………………….…..….2  Gov. Health Center…………...………3  Private Clinic…………………….………4  TBA…………………………………………..5  Traditional Practitioner…………….6  Other (specify) ________________ 7 | | |  |
| 28 | How many times did you receive **scheduled** antenatal care during your last pregnancy**?** | | No. of Times: ______________ 1  Don’t know...............…………..………...2 | | |  |
| 29 | If not four or more times of antenatal care, give reasons why? ***(Explain)*** | | …………………………………………………………  …………………………………………………………. | | |  |
| 30 | During the pregnancy, were you given an injection in the arm to prevent the baby from getting tetanus | | Yes…………………….…………..……1  No……………………………………….2  Don’t remember/Don’t know ………3 | | |  |
| 31 | During antenatal checkup, was the following information given to you? **(Readout)** | | | **Yes** | **No** |  |
|  | 1 | Delivery preparation | | 1 | 2 |  |
|  | 2 | Breastfeeding | | 1 | 2 |  |
|  | 3 | Family Planning (F/P) | | 1 | 2 |  |
|  | 4 | Post Natal Care (PNC) | | 1 | 2 |  |
|  | 5 | Nutrition for self | | 1 | 2 |  |
|  | 6 | Information on HIV/AIDS | | 1 | 2 |  |
|  | 7 | HIV testing | | 1 | 2 |  |
|  | 8 | How to look after you newborn baby | | 1 | 2 |  |
|  | 9 | Importance and use of Insecticide Treated Nets (ITNs) | | 1 | 2 |  |
|  | 10 | The danger signs of pregnancy | | 1 | 2 |  |
|  | 11 | Where to go if you had any of these complications | | 1 | 2 |  |

| 32 | | As part of your ANC during the pregnancy, indicate the check up that was done? | | **Check up** | **Yes** | **No** | | **If yes, how many times** | |  |
| --- | --- | --- | --- | --- | --- | --- | --- | --- | --- | --- |
|  |  |  |  | Weighing | 1 | 2 | |  | |  |
|  |  |  |  | Height | 1 | 2 | |  | |  |
|  |  |  |  | Blood pressure | 1 | 2 | |  | |  |
|  |  |  |  | Blood check | 1 | 2 | |  | |  |
|  |  |  |  | Urine check | 1 | 2 | |  | |  |
|  |  |  |  | HIV Test | 1 | 2 | |  | |  |
| 33 | | What were you told to prepare for delivery ? ***(Circle what is mentioned)*** | | 1. Choose where to deliver from  2. Money saved for transport and hospital needs  3. Gloves  4. Clothes for the baby (cap, socks, vest etc)  5. Polythene/Mackintosh | | | | | |  |
| 34 | During antenatal checkup, were the following given to you? **(Readout)** | | | | **Yes** | | **No** | |  | |
|  | 1 | | Iron tablets | | 1 | | 2 | |  |  |
|  | 2 | | Fansidar | | 1 | | 2 | |  |  |

| **Section 4: Delivery** | | | |
| --- | --- | --- | --- |
| 35 | Where do you give birth from? | At home……………………..…………1  Gov. Hospital…………..………..….2  Gov. Health Center………..……..3  Private Hospital……………………4  Private Clinic…………………..……5  TBA shelter…………………..…….…6  Other (specify)___________ 7 |  |
| 36 | If delivered at home or by TBA, why? | Cost too much at HF…………………..1  Facility not open………………..………2  Facility too far, no transport….…..3  Don’t trust facility services……..…4  Husband did not allow………………5  Not necessary……………………….….6 |  |
| 37 | How did you get to the place of delivery? | Walked………………………………….….1  Bicycle……………………………………...2  Boda Boda…………………………..……3  Motor vehicle…………………..………4  Other specify……………………………5 |  |
| 38 | Who assisted you with transport to the place of delivery? | Husband………………………………….1  Self………………………………………….2  Relative (specify)…………….………3  Friend……….……………………………4  Neighbour…………………..…….……5  Other specify…………………….……6 |  |
| 39 | How was the baby delivered? | Normal delivery…………………...…1  Caesarian section…………..…..…..2  Other (specify)………………………..3 |  |
| 40 | How long after delivery did you stay at place of delivery? | Less than 1 day………………...…….1  1 – 3 days………………………..….….2  More than 3 days…………………….3 |  |
| 41 | What was used to cut the cord during delivery? | Unused / new razor blade……….. 1  Used old razor blade 2  Scissors 3  Knife 4  Other 5  Don’t Know / remember………..…6 |  |
| 42 | What did the attendant use to tie the cord? | Cord bands…………….…………..……1  Cloth strip…………………………...….2  Thread……………………………..….....3  Other (Specify)………………………..4  Don’t know / remember………….5 |  |
| 43 | What major problems did you have related to delivery of the baby?  ***(Multiple response)*** | Bleeding before delivery…….………..…1  Excessive bleeding after delivery…….2  Labor for long time (more 12hrs)…...3  Had Fever….……………………..…………...4  Other (Specify)……………..………..…….5 |  |
| 44 | Was the baby weighed soon after birth? | 1. Yes 2. No |  |
| 45 | How did the spouse assist at the time of delivery? | ……………………………………  …………………………………… |  |

| **SECTION 5: POSTPARTUM CARE (PNC)** | | | |
| --- | --- | --- | --- |
| 46 | After delivery, was the baby wrapped? | 1. Yes 2. No |  |
| 47 | If yes, how soon was the baby wrapped | ……………………….hrs |  |
| 48 | If yes, what was used to wrap the baby? ***(Explain)*** | Blanket……………………………….…..1  Towel………………………………………2  Mother’s cloth………………………..3  Sheet………………………………..…….4  Other (specify)..………………………5 |  |
| 49 | Did you ever breastfeed the baby? | Yes…………………………….………….1  No………………………………….……..2 |  |
| 50 | Did you give the baby the first milk (colostrums) that came from your breasts? | Yes…………………….………………….1  No……………………….………………..2 |  |
| 51 | If no, why didn’t you give the baby the first milk that came from your breasts?  *(explain)* | ……………………………………  ……………………………………  …………………………………… |  |
| 52 | Did you apply anything on the baby cord after delivery? | Yes……………………………………….1  No………………………………………..2 |  |
| 53 | If yes, what did you apply to care for the baby’s cord? | Cleaned with spirit….………….. 1  Put Vaseline…………………….... 2  Put herbs……………..……………..3  Put non-herbal mixtures....... 4  Put nothing………………………...5  Baby powder………………………6  Other (specify) __ 7 |  |
| 54 | Did the eyes get any discharge during first months of life? | Yes………….……………………….1  No……………………………………2 |  |
| 55 | If yes, what did you do to care for the discharging eyes?*(explain)* | ……………………………………  ……………………………………  ……………………………………  …………………………………… |  |
| 56 | What signs / symptoms would make you seek treatment outside home for young infant less than one month?  ***(MULTIPLE RESPONSE)***  ***(Don’t read for respondent)*** | Poor sucking /not feeding…………….….1  Difficult or fast breathing.. …………….. 2  Red swollen eyes with discharge….....3  Redness and discharge around cord…4  Skin lesions (or blisters)………………..…5  Abnormal body swellings……...….….…6  Yellow skin/eye color (jaundice) ........7  Red cord ………………………………………...8  Palm/sores/mouth blue (cynosis)…. 9  Cold/shivering (hypothermia)………..10  Convulsions/twitches.……… . …........11  Bulging fontanelle…………….……….....12  Persistent/abnormal cry…………….….13  Rigidity of body ………………………..… 14  Vomiting/regurgitation……………...…15  Frequent watery stools/or stools… .16  Failure to pass stools………..………... ..17  Failure to pass urine……………………..18  Peeling skin…………………………….…...19  Other (specify) _______________20  Don’t know……………………...............21 |  |
| 57 | In your opinion who is the **main** person who makes decisions relating to the care of your newborn. | Health worker…………………………...1  Paternal grandmothers …………….2  Maternal grandmothers ……………3  Husbands/Partners ………………… 4  Mothers of newborns…………..…….5  Other _ 6 (Specify) |  |

| **SECTION 6: ATTITUDE & BELIEFS** | | | |
| --- | --- | --- | --- |
| 58 | What is your opinion about health facility deliveries ? | Very important …………………….………1  some what important …………………...2  waste of time …………………………….….3  Any other (specify) …………………………4 |  |
| 59 | What are some of benefits of antenatal care? |  |  |
| 60 | What are some of the benefits of health facility deliveries ? |  |  |
| 60 | Do you know people in this community that do not deliver their babies in the health facility? | Yes………………………………….1  No…………………………………..2 |  |
| 61 | Why do you think they do not have health facility deliveries ? | 1. Religious beliefs (specify) ………………………………………………………..   ………………………………………………………..   1. Traditional beliefs (specify)…………………   ………………………………………………………….  ………………………………………………………….   1. Mother was sick 2. Long distance to health centers 3. Lack of permission from the spouses 4. Not pleased with antenatal care services offered at the health centre 5. Advised by their peers 6. Time of antenatal care – not convenient 7. Other (specify)…………………………………. |  |

| **SECTION 7: SOURCES OF INFORMATION** | | | |
| --- | --- | --- | --- |
| 62 | In this community, what are your sources of information about health facility deliveries? | 1. radio  2. friends  3. health worker  4. family members  5. newspapers  6. other specify ……………………………… |  |
| 63 | Have you heard someone in this community talking against health facility deliveries? (please tick ) | Yes………………………………….1  No…………………………………..2 |  |
| 64 | If “Yes” Indicate some of the beliefs you have heard concerning health facility deliveries. | ……………………………………………………………  …………………………………………………………… |  |
| 65 | Who in the community can best convince expectant mothers about health facility based deliveries? | 1. Health worker  2. Local Council  3. Family members  4. Friends  5. Church leaders  6. any other specify ……………………………… |  |

1. Which of the following methods could be effective in convincing mothers to have health facility deliveries ? *(please tick the correct cell)*

|  |  | Very Important | Important | Not Important |
| --- | --- | --- | --- | --- |
| 1 | Continuous health education and sensitization |  |  |  |
| 2 | Show education films and photos of effects of poor neonatal healthcare and deliveries |  |  |  |
| 3 | Continuous radio announcements |  |  |  |
| 4 | Booklets, brochures with pictures, illustrations translated in local languages to be continuously be given |  |  |  |
| 5 | Involvement of health workers and local council members in moving around, talking to mothers/women. |  |  |  |
| 6 | Motivation of those who attend hospital deliveries. |  |  |  |
| 7 | Others (specify) ……….………………………… |  |  |  |

| **SECTION 8: QUALITY OF HEALTHCARE SERVICES** |
| --- |

1. Rate the following services *(* 5= very good, 4=good, 3=fair, 2 = bad 1=very bad)

|  |  | Very good | Good | Fair | Bad | Very Bad |
| --- | --- | --- | --- | --- | --- | --- |
| 1 | The attitude of the service providers (health workers |  |  |  |  |  |
| 2 | The equipment used (syringes, cotton wool) |  |  |  |  |  |
| 3 | The health facility/outreach environment (hygiene, convenient) |  |  |  |  |  |
| 4 | Days and time of antenatal care |  |  |  |  |  |

67. In your opinion, state the importance of the following in encouraging expectant mothers to attend antenatal services :

|  |  | Very Important | Important | Not Important |
| --- | --- | --- | --- | --- |
| 1 | Home visiting to be done by health worker |  |  |  |
| 2 | Health education sessions while waiting for antenatal care and allowing time for questions |  |  |  |
| 3 | Good attitude, being approachable, kind, friendly, polite health workers |  |  |  |
| 4 | Put up notices of antenatal service days and time by the road sides |  |  |  |
| 5 | Social mobilization / outreach facilities |  |  |  |
| 6 | Health workers attending village meetings of the neighboring places and sharing information |  |  |  |
| 7 | Reward expectant mothers who attend ANC. |  |  |  |

68. State which of the following needs to be improved at the health facility where you go for antenatal care.

|  |  | Greatly needed | Needed | Not Needed |
| --- | --- | --- | --- | --- |
| 1 | Number of health workers at the health facility to avoid keeping expectant mothers waiting |  |  |  |
| 2 | Logistics (cotton wool, syringes) |  |  |  |
| 3 | More time for questions during antenatal care session |  |  |  |
| 4 | Hygiene of the health facility |  |  |  |
| 5 | More days and time antenatal services |  |  |  |
| 6 | Improvement in availability of drugs |  |  |  |
| 7 | Other (specify)…………………… |  |  |  |

69. State how important the following can be utilized by community leaders (Religious leaders, LC Leaders, Women Leaders, Political Leaders) to disseminate correct messages about antenatal care services?

|  |  | Very Important | Important | Not Important |
| --- | --- | --- | --- | --- |
| 1 | By inviting health workers to sensitize the community during LC Meetings, ladies meetings, church meetings etc. |  |  |  |
| 2 | Political leaders can continuously educate the community when rallies are held |  |  |  |
| 3 | Others (specify) ………………………………… |  |  |  |

***Thank you very much for your time and co-operation.***

***The information you have given me is valuable for the program.***

**FRONTLINE HEALTH WORKER INTERVIEW GUIDE**

This interview guide is designed to facilitate a study that investigates the factors associated with maternal and neonatal healthcare. Your opinion is very important and will help in suggesting ways that would help improve maternal and neonatal healthcare services.

1. **GENERAL INFORMATION**
2. Name of health facility (optional) ………………………………………………………………………………….
3. Indicate the type of health facility (please tick).

| Health Centre III |  |  | Referral Hospital |  |
| --- | --- | --- | --- | --- |
| Health Centre IV |  |  | Private Hospital |  |
| Private Health Centre |  |  | Any other …………………………….. |  |

1. Parish ……………………………………………………………………………………………………………………….
2. District ……………………………………………………………………………………………………………………..
3. Indicate the number of health workers at the health facility (please tick).

| Gynecologist |  |  | Midwives |  |
| --- | --- | --- | --- | --- |
| Pediatrician |  |  | Nurses |  |
| Other consultants |  |  | Lab technicians |  |
| Medical Doctors |  |  | Pharmacy technicians |  |
| Medical Assistants |  |  | Non-skilled support staff |  |

1. Indicate the services available at the health facility (please tick).

| Maternity |  |  | Pharmacy |  |
| --- | --- | --- | --- | --- |
| Paediatric |  |  | x-ray |  |
| Laboratory |  |  | Ultra Sound Scan |  |
| Family Planning |  |  | Caesarean section |  |
| Other………………………. |  |  | Other ……………………………………… |  |

1. Indicate how many deliveries are handled every month (please tick).

| 0 - 10 |  |  | 31-40 |  |
| --- | --- | --- | --- | --- |
| 11-20 |  |  | 41-50 |  |
| 21-30 |  |  | 50+ |  |

1. State whether the following are available and observed by you at the health facility.

|  |  | Always | Often | Never |
| --- | --- | --- | --- | --- |
|  | Health facility is open 24 hours every day including skilled and non skilled health workers |  |  |  |
|  | Cleanliness of the care areas; debris/trash, walls, ceiling |  |  |  |
|  | Presence of a checklist for identifying sick newborns |  |  |  |
|  | Poster with listed services in both English and local language displayed in the waiting areas where clients can see |  |  |  |
|  | Criteria for referral in case the condition is beyond what the health facility can handle. |  |  |  |
|  | Referral notes (slip with diagnosis) given to women |  |  |  |
|  | Sick newborns and mothers are visited at least once by the a doctor or clinical officer |  |  |  |

1. Indicate how long clients wait for family planning services, antenatal care and labour at the health facility before being attended to.
2. 0-30 minutes b) 31-60 minutes c) more than 1 hour
3. **INFRASTRUCTURE AND EQUIPMENT**
4. Indicate the infrastructure for newborn health services available at the health facility? State their condition.

|  |  | Please tick | **Condition**  **Working or Not Working** |
| --- | --- | --- | --- |
|  | Theater |  |  |
|  | Resuscitation table |  |  |
|  | Post natal ward |  |  |
|  | Paediatric ward |  |  |
|  | Nursery space close to labour ward |  |  |
|  | Beds for Kangaroo Mother Care |  |  |
|  | Incubators for pre-term babies |  |  |
|  | Phototherapy |  |  |
|  | Any other ……………… |  |  |

1. Indicate the equipment for newborn health services available at the health facility? State their condition.

|  |  | Please tick | Condition  Working or Not Working |
| --- | --- | --- | --- |
|  | Thermometer |  |  |
|  | Infant Ambu bags and masks |  |  |
|  | C-Section equipment |  |  |
|  | Baby weighing scale |  |  |
|  | Baby oropharyngeal airway |  |  |

1. Indicate the supplies for newborn health services available at the health facility?

|  |  | Please tick |
| --- | --- | --- |
|  | Cannulas, NG tubes |  |
|  | Baby syringes |  |
|  | Single use bulb syringe |  |
|  | Swabs |  |
|  | Gloves |  |
|  | Feeding cups for small babies |  |
|  | Blood, IV Fluids – normal saline, dextrose ringer lactate |  |
|  | Delivery kits with (2 clean drape, new blades, 2 clean cord clamp, 2 pairs of gloves) assembled and accessible for use. |  |

1. Indicate whether you observe the following check lists at the health facility?

|  |  | Please tick |
| --- | --- | --- |
|  | Checklist for new born equipment |  |
|  | Check list for new born supplies |  |

1. **CLINICAL SERVICES**
2. Indicate the maternal care services provided in this health facility

|  |  | | Always | Often | Never |
| --- | --- | --- | --- | --- | --- |
|  | | Screening for STI infection in antenatal care |  |  |  |
|  | | Screening for HIV infection in antenatal care |  |  |  |
|  | | Screening for Malaria infection in antenatal care |  |  |  |
|  | | Assessment for mothers’ weight , blood pressure |  |  |  |
|  | | Assessment for mothers’ anemia |  |  |  |
|  | | Monitoring labour with partograph |  |  |  |
|  | | 4 hourly vagina examination |  |  |  |
|  | | Medical pre-operative review for anticipated critical events by doctors and nursing team |  |  |  |
|  | | Diabetes during pregnancy |  |  |  |

1. Indicate the newborn care inpatient services observed after delivery in this health facility

|  |  | | Always | Often | Never |
| --- | --- | --- | --- | --- | --- |
|  | | Assessment of baby every 4 hours for breathing |  |  |  |
|  | | Assessment of baby every 4 hours for feeding |  |  |  |
|  | | Assessment of baby every 4 hours for warmth |  |  |  |

1. Indicate the newborn care specialised services offered in this health facility

|  |  | | Yes | No |
| --- | --- | --- | --- | --- |
|  | | Phototherapy |  |  |
|  | | Continuous Positive Airway Pressure |  |  |
|  | | Blood transfusion |  |  |
|  | | Post natal care |  |  |
|  | | Caring for low birth weight babies |  |  |

1. What clinical services are observed before a newborn is discharged?

|  |  | | Always | Often | Never |
| --- | --- | --- | --- | --- | --- |
|  | | Newborn stays with the mother in the health facility for minimum of 24 hours |  |  |  |
|  | | Mother receives education on cord care, warm chain and breast feeding |  |  |  |
|  | | Mothers informed on danger signs to watch out for at home |  |  |  |
|  | | Mother given post natal appointments |  |  |  |

1. **INFECTION PREVENTION**
2. Indicate which facilities for infection control and disinfection are available at the health facility

|  |  | | Please tick |
| --- | --- | --- | --- |
|  | | Soap and water at washing points, near examination room, labor ward , theatre, nursery, pediatric and postnatal wards |  |
|  | | Sterilizer |  |
|  | | Disinfectant |  |
|  | | Clean packaging and storage in delivery, theatre, pediatric and nursery rooms |  |
|  | | A bucket with disinfectant and functional sterilization mechanism |  |
|  | | Safety boxes and waste bins |  |

1. State the infection control practices observed at the health facility (only tick those mentioned)

|  |  | | Please tick |
| --- | --- | --- | --- |
|  | | Hand washing with soap and water between examinations |  |
|  | | Hand washing with soap and water before feeding the baby |  |
|  | | Hand washing with soap and water after changing baby’s nappy |  |
|  | | Hand washing with soap and water before and after wound dressing |  |
|  | | Hand washing with soap and water before and after giving injections and IV fluids |  |
|  | | Safe disposal of sharp objects |  |

1. **INFORMATION, EDUCATION AND COMMUNICATION**
2. Indicate the issues the clients are educated on during the antenatal, post natal and family planning clinics visit.

|  |  | Please tick |
| --- | --- | --- |
| a. | HIV testing, mother to child transmission |  |
| b. | Danger signs during pregnancy |  |
| c. | Infant and young child feeding |  |
| d. | Cord care |  |
| e. | Extra care for small babies |  |
| f. | Personal hygiene |  |

1. Indicate the materials available during client counseling / education at the health facility

|  |  | Please tick |
| --- | --- | --- |
| a. | Posters |  |
| b. | Child spacing methods |  |
| c. | Brochures / Leaflets |  |
| d. | Flip charts |  |

1. **MANAGEMENT SYSTEMS**
2. Indicate the records used at this health facility? (Check for availability)

|  |  | Please tick | Briefly state how they are used |
| --- | --- | --- | --- |
| a. | Antenatal, birth and post natal registers |  |  |
| b. | Client/patient case management notes |  |  |
| c. | Partographs |  |  |
| d. | Discharge / referral forms |  |  |
| e. | Death Certificate |  |  |
| f. | Perinatal death audit books |  |  |
| g. | Child health cards |  |  |
| h. | Completed HMIS forms |  |  |
| I | Peri/neonatal audit reports |  |  |
| j | Treatment and Monitoring chart of the sick newborn |  |  |

1. Indicate the essential medicines/emergency drugs available at this health facility? (Check for availability)

|  |  | Please tick |
| --- | --- | --- |
| a. | Ampicillin |  |
| b. | Gentamycin |  |
| c. | Multivitamins |  |
| d. | Iron syrup |  |
| e. | Vitamin K |  |
| f. | Tetracyline Eye Ointment |  |
| g. | I.V. Fluids Dextrose |  |
| h. | Oxygen |  |
| I | Adrenalin |  |
| j | Phenobarbitone |  |

1. Does the health facility carry out Kangaroo Management Care (KMC) ?

a) Yes b) No

1. If the answer is “Yes”, indicate whether the following are available at the health facility.

|  |  | Please tick |
| --- | --- | --- |
| a. | Skilled health workers in managing KMC |  |
| b. | KMC bed in post natal ward |  |
| c. | Designated space for KMC |  |
| d. | KMC wrappers for demonstration |  |

1. **GUIDELINES**
2. Does the health facility have standard neonatal guidelines ?

a) Yes b) No

1. If “Yes”, where are these guidelines kept ? ……………………………………………………………….....................

…………………………………………………………………………………………………………………………………………

1. Do you carry out perinatal and maternal audits? ………………………………………………………………………..
2. Briefly describe what is done during the audits ……………………………………………………………………………

…………………………………………………………………………………………………………………………………………

1. How do you use the information obtained from the audits ?..............................................

…………………………………………………………………………………………………………………………………………

1. **PROBLEMS ENCOUNTERED**
2. Have you encountered a death experience at this health facility ?
3. Yes b) No
4. If the answer for Question 31 is “Yes”, indicate the causes of death of neonates and how frequently they occur.

|  | Cause of Death | Frequently occur | Rarely occur | Never occur |
| --- | --- | --- | --- | --- |
| 1 | Due to mother’s labour complications |  |  |  |
| 2 | Neonatal infections |  |  |  |
| 3 | Pre-term and low birth weight |  |  |  |
| 4 | Hypothermia |  |  |  |
| 5 | Congenital malformation |  |  |  |
| 6 | Others (specify) ……………………………………. |  |  |  |

1. Indicate the problems encountered at the health facility ?

|  | **Category** | **State the problem** | **Specify what needs to be improved** |
| --- | --- | --- | --- |
|  | Clients /Patients |  |  |
|  | Human Resources |  |  |
|  | Drugs, vaccines, supplies availability |  |  |
|  | Laboratory services |  |  |
|  | Ambulances/ Transportation |  |  |
|  | Electricity |  |  |
|  | Water (safe and clean) |  |  |
|  | Mobilisation of mothers |  |  |
|  | Health policies and guidelines |  |  |

1. **IMPROVEMENTS AT THE HEALTH FACILITY**
2. Which of the following needs to be improved at the health facility ?

|  |  | **Please tick** | **Specify what needs to be improved** |
| --- | --- | --- | --- |
| 1 | Delivery Equipment |  |  |
| 2 | Neonatal Equipment |  |  |
| 3 | Drugs, vaccines, supplies availability |  |  |
| 4 | Laboratory services |  |  |
| 5 | Ambulances/ Transportation |  |  |
| 6 | Electricity |  |  |
| 7 | Water (safe and clean) |  |  |
| 8 | Mobilisation of mothers |  |  |
| 9 | Health workers |  |  |
| 10 | Staff remuneration |  |  |
| 11 | Other (specify)  ……………………………………… |  |  |

**Thank you very much for your time and cooperation.**

**VILLAGE HEALTH WORKER INTERVIEW GUIDE**

This interview guide is designed to facilitate a study that investigates the factors associated with maternal and neonatal healthcare. Your opinion is very important and will help in suggesting ways that would help improve maternal and neonatal healthcare services.

1. **GENERAL INFORMATION**
2. Name of the village health worker (optional) ……………………………………………………………………………
3. Title / Training of the village health worker (please tick)

| Village Health Worker |  |  | Traditional Birth Attendant |  |
| --- | --- | --- | --- | --- |
| Retired Midwife |  |  | Retired Nurse |  |
| Any other |  |  | Any other ……………………………… |  |

1. Village …………………………………………………………………………………………………………………………………
2. Parish …………………………………………………………………………………………………………………………………..
3. District …………………………………………………………………………………………………………………………………
4. Indicate the health facility you are attached to ……………………………………………………………………
5. How long have you worked as a Village Health Worker

a) 0-2 years b) 3-4 years c) 5 + years

1. On average indicate how many deliveries you attend to every month (please tick).

| 0 - 5 |  |  | 16-20 |  |
| --- | --- | --- | --- | --- |
| 6-10 |  |  | 21-25 |  |
| 11-15 |  |  | 25+ |  |

1. Has any of your clients lost their newborns during your career ? …………………………….
2. Yes b) No
3. If the answer for Question 9 is “Yes”, indicate the causes of death of neonates and how frequently they occur.

|  | Cause of Death | Frequently occur | Rarely occur | Never occur |
| --- | --- | --- | --- | --- |
| 1 | Due to mother’s labour complications |  |  |  |
| 2 | Neonatal infections |  |  |  |
| 3 | Pre-term and low birth weight |  |  |  |
| 4 | Hypothermia |  |  |  |
| 5 | Congenital malformation |  |  |  |
| 6 | Others (specify) ……………………………………. |  |  |  |

1. **CARE DELIVERY PROVIDED**
2. Indicate on which days you visit mothers and newborns after delivery (please tick)

|  |  | Please tick |
| --- | --- | --- |
|  | Day 1 (after 24 hours after birth) |  |
|  | Day 3 |  |
|  | Day 7 |  |
|  | Additional visit on day 5 and 10 for small babies |  |
|  | No visit at all |  |

1. Indicate the advice you give to mothers to keep the babies warm (Do not read these)

|  |  | Please tick |
| --- | --- | --- |
|  | Drying |  |
|  | Wrapping baby |  |
|  | Skin to skin contact to maintain warmth |  |
|  | Delay bathing for 24 hours |  |
|  | Kangaroo care for small babies |  |

1. Indicate the advice you give to mothers to keep the cords clean

|  |  | Please tick |
| --- | --- | --- |
|  | Keep cord dry and clean |  |
|  | Put nothing on it |  |
|  | Put saline solution |  |
|  | Put spirit |  |
|  | Any other …………………………………………………………… |  |

1. Indicate the advice you give to mothers concerning feeding the newborn babies

|  |  | Please tick |
| --- | --- | --- |
|  | Initiate breastfeeding within one hour |  |
|  | Exclusive breastfeeding |  |
|  | Chosen feeding method if mother is HIV+ |  |
|  | Any other …………………………………………………………… |  |

1. Indicate the advice you give to mothers concerning taking the newborns to health facilities

|  |  | Please tick |
| --- | --- | --- |
|  | Take the babies for immunisation |  |
|  | Take babies for HIV testing |  |
|  | Encourage the use of ITN |  |

1. Indicate the advice (reasons) you give to mothers concerning visiting the health facilities after birth

|  |  | Please tick |
| --- | --- | --- |
|  | Post natal checks |  |
|  | Child spacing (family planning services) |  |
|  | Any other ………………………………………………………………….. |  |
|  |  |  |

1. **RECORD MANAGAMENT**
2. Are you supplied with village registers and mother baby care education materials?
3. Yes b) No
4. Who supplies you with village registers and mother baby care education materials?

……………………………………………………………………………………………………………………………………..

1. Indicate the records you keep during the village visits

|  |  | Please tick |
| --- | --- | --- |
|  | Births and deaths |  |
|  | Post natal visit findings |  |
|  | Referred cases |  |
|  | Any other …………………………………………………………… |  |

1. Are the records incorporated or linked to the health facility HMIS data?
2. Yes b) No
3. If the answer for Question 20 is “Yes”, indicate how often this is done in a year (please tick)

| Once |  |  | Thrice |  |
| --- | --- | --- | --- | --- |
| Twice |  |  | 4 times and more |  |

1. Do the health facility visit you to check/supervise your healthcare activities?
2. Yes b) No
3. If the answer for Question 22 is “Yes”, indicate how often this is done in a year (please tick)

| Once |  |  | Thrice |  |
| --- | --- | --- | --- | --- |
| Twice |  |  | 4 times and more |  |

1. Do you hold meetings with health facility staff?
2. Yes b) No
3. If the answer for Question 24 is “Yes”, indicate how often this is done in a year (please tick)

| Once |  |  | Thrice |  |
| --- | --- | --- | --- | --- |
| Twice |  |  | 4 times and more |  |

1. **PROBLEMS ENCOUNTERED AND POSSIBLE SOLUTIONS**
2. Indicate the problems related to maternal and newborn health you encounter during your work as a Village Health Worker as well as possible solutions.

|  | **State the problem** | **Possible Solutions** |
| --- | --- | --- |
|  |  |  |
|  |  |  |
|  |  |  |
|  |  |  |
|  |  |  |

**Thank you very much for your time and cooperation.**

**District and Community Leaders interview guide**

**Leaders (Secretary for social services, CAO, LC1, Secretary for women, Chairman health committee at LC IV or LC III)**

District______________________________

Sub County__________________________

1. **Knowledge and roles**
2. What do you know about the health of newborns and their mothers?

……………………………………………………………………………………

1. How are you involved in issues of health of newborn and that of their mothers? (probe: Registration of birth, death, mobilization etc)

……………………………………………………………………………………

1. **Programs**
2. What opportunities exist at your level of function to improve newborn health and that of their mother?

……………………………………………………………………………………

1. Do you know of any programmes that are targeting the health of newborns and mothers?

……………………………………………………………………………………

1. What is good about services offered in the health units for the newborn and their mothers?

……………………………………………………………………………………

1. What is not so good about services offered in the health units for the newborn and their mothers?

……………………………………………………………………………………

1. How can communities and households be mobilized to improve health of newborn and their mothers?

……………………………………………………………………………………

1. **Suggestions for improvement**
2. In your view, what should be done to improve the health of newborns and that of their mothers;
   1. At household……………………………………………………
   2. Community…………………………………………………………

10. What suggestions do you have for improving the health of newborns and their mothers?

1. Before pregnancy…………………………………………………………
2. During pregnancy…………………………………………………………
3. Maternity (Labor and delivery)………………...…………………………
4. After delivery……..………………………………………………………
5. Do you have any questions to ask about newborn and mothers?

……………………………………………………………………………………

# Thank you

## HEALTH POLICY IMPLEMENTERS INTERVIEW GUIDE

This interview guide is designed to facilitate a study that investigates the factors associated with maternal and neonatal healthcare.

District______________________________

Sub County__________________________

Title _______________________________

**NEWBORN CARE**

1. s newborn care a prioritized area of service delivery in the district?
2. What are the current programme activities for the newborn in the district?
3. What are the key roles played by the different stakeholders at various levels? (Probe: planning, logistics, service delivery and reporting).
4. Is Newborn care reflected in district plans?
5. What is the human resource capacity for newborn care in the district?
6. Has the district organize in service training for newborn care in the last two years
7. Is there any special funding for the Newborn, if some what proportion to the health budget?

**CARE FOR THE MOTHERS**

1. a) What is the estimated coverage of F/P, ANC, basic obstetric care (maternity) and EoMC in your district?

b) How has this changed over the years? *(Possible reasons for the trend)*

c) How adequate are the services provided? (Availability of supplies e.g. vaccines, needles, full service package delivery)

d) What mechanisms are in place for monitoring and support supervision at lower levels

e) What are the major challenges for providing this service in the district?

9. What are the opportunities to improve ANC services in the district?

1. What percentage of mothers deliver in health units
2. What are the major reasons for mothers not delivering at home
3. What opportunities exist in the district to get more mothers deliver in health units
4. For mothers that deliver in health units, how long should they stay in the units?
5. What services are given in the immediate postpartum period for mothers delivering in hospital rather than admitted.

**CHALLENGES, OPPORTUNITIES AND RECOMMENDATIONS**

15. What are the major challenges of reducing newborn and maternal mortality in the district?

1. What opportunities exist to reduce newborn and maternal mortality in the district?
2. Make specific recommendations to improve services to reduce newborn death in the district.
3. Do you have any other questions or concerning newborn health and care?.

# Thank you
