# Supplementary material for: Advancing the application of systems thinking in health: understanding the dynamics of neonatal mortality in Uganda
Source: Health Res Policy Syst. 2014 Aug 8;12:36. doi: 10.1186/1478-4505-12-36 (PMC4134459; doi:10.1186/1478-4505-12-36)
Supplement: Additional file 2 — Sample size calculation. [file 1478-4505-12-36-S2.docx]

Additional file 2: Sample size estimation for interviews with mothers

For the purposes of determining the sample size, it was assumed that the population was well deﬁned and there were no systematic biases in the selection of mothers that could adversely inﬂuence the conclusions. At neonatal death rate (p) of 5.4%, 95% confidence interval and level of permissible error (e) as e ≤ 5%, the sample size n was determined by the following equation:

|  | $n=\frac{z^{2}\mathrm{pq}}{e^{2}}$ | *(Eq.1)* |
| --- | --- | --- |

where:

- n is the sample size,
- Z^2^ is the standard normal deviate corresponding to 95% confidence (1.96),
- p is the estimated percentage of neonates who die 5.4% (UDHS 2011)
- q is 1-p.ie 96.4%
- e is the desired level of precision which was taken as 5%
- Design effect of 1.5
- An addition of 20% loss to non-response was added.

The design factor of 1.5 was incorporated to adjust the standard error resulting from the following design features: stratification of the sample to guarantee that sub-groups of mothers selected from homes and health facilities appear in the correct proportions ([proportionate stratification](http://www.restore.ac.uk/PEAS/sratheory.php#explicit)), and weighting of the sample to adjust for non-equal probabilities of selection and [non-response](http://www.restore.ac.uk/PEAS/nonresponse.php).
