# Supplementary material for: Advancing the application of systems thinking in health: understanding the dynamics of neonatal mortality in Uganda
Source: Health Res Policy Syst. 2014 Aug 8;12:36. doi: 10.1186/1478-4505-12-36 (PMC4134459; doi:10.1186/1478-4505-12-36)
Supplement: Additional file 3 — List of variables. [file 1478-4505-12-36-S3.docx]

# Additional file 3: List of Variables

Table 1: Health service factors associated with the mothers’ attendance to health services

| **Theme** | **Factors associated with attendance to health services** | **Existing evidence** | **Source from field studies** | **Observed in Field Studies (Y/N)** | **Effect** |
| --- | --- | --- | --- | --- | --- |
| **Health Service** | Quality care at the health facility | Eijk *et al*.,( 2006); Marsh *et al*. (2002); Turkson (2009) | Mothers’ interviews | Y | Positive |
|  | Insufficient number of trained health workers. |  | Mothers’  FHW interviews | Y | Negative |
|  | Lack of supplies, basic equipment and essential drugs | MOH(2008); MNCH (2006); Sharma, 2012; Dzadeyson, 2007; Lanre-Abass (2008) | Mothers’  FHW interviews | Y | Negative |
|  | Lack of water, electricity supply at the health facility | Lanre-Abass (2008) | Mothers, FHW Interviews | Y | Negative |
|  | Long waiting times at the health facility | Turkson (2009) | Mothers’ Interviews | Y | Negative |
|  | Inconvenient and insufficient time given to mother during ANC session |  | Mothers’  Interviews | Y | Negative |
|  | Congestion in the health facility |  | Mothers’  FHW Interviews | Y | Negative |
|  | Waiting times for mothers resulting in frustration |  | Mothers’ Interviews | Y | Negative |
|  | Unfriendly staff attitudes to patients | Lanre-Abass (2008) | FHW Interviews | Y | Negative |
|  | Myths e.g. health workers steal babies |  | Mothers’ Interviews | Y | Negative |

Table 2 Social / Personal Characteristics associated with mothers’ attendance to health services

| **Theme** | **Factors associated with attendance to health services** | **Existing evidence** | **Source from Field Studies** | **Observed / Field Studies (Y/N)** | **Effect** |
| --- | --- | --- | --- | --- | --- |
| **Social / Personal**  **factors** | Poor social economic status (affordability) | WHO (2006); MOH(2008); Uddin and Hossain (2010); Chowdhury *et al*., (2010) ; Kumar and File (2005) | FHW,  Mothers’ interviews | Y | Negative |
|  | Low level of maternal education | MOH (2008); Katahoire (1998); UBOS & ORC-Macro (2006); Baral *et al*. (2012). | FHW,  Mothers’  interviews | Y | Negative |
|  | Low level of paternal education | Chowdhury *et al.,* (2010); Baral *et al*. (2012); Adamu and Salihu (2002) | Not captured | N | Negative |
|  | Area of residence (impoverished) | Uddin and Hossain (2010); UDHS (2011); Baral *et al*. (2012) | Not captured | N | Negative |
|  | Maternal age (younger than 30 years) | MOH(2008); Mondal *et al.*(2009); Chowdhury *et al*., (2010); Baral *et al*. (2012) | Mothers’  interviews | Y | Negative |
|  | Poor accessibility to health facility ( | Lanre-Abass (2008); Eijk *et al*.,( 2006) | Mothers’  interviews | Y | Negative |
|  | Permission from work place | Lanre-Abass (2008) | Mothers’ interviews | Y | Positive |
|  | Permission from spouse | Lanre-Abass (2008) | Mothers’ interviews | N | Positive |
|  | Mother’s busy schedule | Uddin and Hossain (2010); Chowdhury et al., (2010) | Mothers’ interviews | Y | Negative |
|  | Traditional/Religious beliefs | Adamu and Salihu (2002) | Mothers’ interviews | Y | Negative |
|  | Access and utilization of reproductive services (family planning) | Lanre-Abass (2008) | Mothers’ Interviews | N | Positive |
|  | Socially unstable (single parent/unmarried) |  | Mothers’ interviews | N | Negative |
|  | Big number of living children (more than 4) | Baral *et al*. (2012) | Mothers’ Interview | N | Negative |
|  | Trust in healthcare service | Turkson (2009) | Mothers’ interviews | Y | Positive |
|  | Availability of Immunisation services for infants | Mondal *et al.*(2009) | Mothers’ interviews | Y | Positive |
|  | Ignorance and belief that they can delivery without the help of health worker |  | Mothers’  FHW Interviews | Y | Negative |

Table 3 Community/Family Factors associated with the mothers’ attendance to health services

| **Theme** | **Factors associated with mothers’ attendance to health services** | **Existing evidence** | **Source from field studies** | **Observed / Field Studies (Y/N)** | | **Effect** |
| --- | --- | --- | --- | --- | --- | --- |
| **Family/** | Husband’s acceptance |  | Not captured | | N | Positive |
| **Community** | Gender based violence | Marsh *et al.* (2002) | Mothers’ interviews | | N | Negative |
|  | Family (spouse) support – provision of transport | MNCH (2006) |  | | Y | Positive |
|  | Community support by providing care and transport |  | Mothers’ Interviews | | Y | Positive |
|  | Involvement of Community Leaders (political, religious) in mobilization of mothers | MNCH (2006) | Mothers’ Interviews | Y | | Positive |
|  | Peer to peer influence through discussions with other mothers |  | Mothers’ Interviews | Y | | Positive |

Table 4 Factors associated with neonatal health

| **Theme** | | **Factors associated with neonatal health** | **Existing evidence** | **Source from field studies** | **Observed / Field Studies (Y/N)** | | **Effect** |
| --- | --- | --- | --- | --- | --- | --- | --- |
| **Health of the mothers** | | Feeding and nutrition | MNCH (2006); Chowdhury *et al.,* (2010) | FHW  Mothers’ interviews | N | | Positive |
|  | | Hygiene and household environment | Uddin and Hossain (2010); MOH (2008); MNCH (2006); Sharma (2012); Dzadeyson (2007) | FHW  Mothers’ interviews | N | | Positive |
|  | | Frequent deliveries, child interval (spacing) | Mondal *et al.*(2009); Chowdhury *et al.,* (2010); Kumar and File (2005) | FHW  Mothers’ interviews | N | | Negative |
|  | Poor breastfeeding practices | | Chowdhury *et al*., (2010); Marsh *et al*., 2002; MOH (2008); MNCH (2006); Sharma (2012); Dzadeyson (2007) | Mothers’ interviews | | N | Negative |
|  | | Untreated diseases (Fever, Malaria) | Zadkaarami (2008); MOH(2008); MNCH (2006); Ndyomugenyi, Neema, &Magnussem (1998); Hong (2006) | FHW  Mothers’ interviews | | Y | Negative |
|  | | Long labour hours during delivery |  | FHW  Mothers’ Interviews | Y | | Negative |
|  | | Excessive bleeding during delivery |  | FHW,  Mothers’ Interviews | Y | | Negative |
|  | | Gender based violence |  | Mothers’ interviews | N | | Negative |
| **Neonates** | | Premature and low birth weight of baby | Marsh *et al*., 2002; MOH(2008); MNCH (2006); Sharma, 2012; Dzadeyson, 2007 | Mothers’ Interviews | Y | | Negative |
|  | | Child’s illness |  | Mothers’ Interviews | Y | | Negative |
|  | | Hypothermia | Byaruhanga, Berishom and Okong(2005); MOH(2008) | Mothers’ Interviews | Y | | Negative |
| **Knowledge**  **Beliefs and**  **Attitudes** | | Lack of knowledge about the care of neonates such as care of the cord and eyes and maternal care. | Midhet and Becker (2010); Uddin and Hossain (2010) | Mothers’ interviews | | Y | Positive |
|  | | Belief in myths and perceptions | MNCH (2006); Marsh *et al*., (2006) | Mothers’ interviews | | Y | Negative |

Table 5 Factors associated with the service delivery in health facilities

| **Theme** | **Factors associated with health service delivery in health facilities** | **Existing evidence** | **Source from field studies** | **Observed / Field Studies (Y/N)** | **Effect** |
| --- | --- | --- | --- | --- | --- |
| **Health Service** | Lack of quality care at the health facility | Eijk *et al*.,( 2006); Turkson (2009) | Mothers, FHW Interviews | Y | Negative |
|  | Long waiting times | Turkson (2009) | Mothers’ Interviews | Y | Negative |
|  | Lack of diagnostic and resuscitation kits, basic equipment and essential drugs | MOH(2008); MNCH (2006); Sharma, 2012; Dzadeyson, 2007; Lanre-Abass (2008) | Mothers, FHW Interviews | Y | Negative |
|  | Lack of water, electricity supply at the health facility | Lanre-Abass (2008) | Mothers, FHW Interviews | Y | Negative |
|  | Staff burnout due to workload |  | FHW Interviews | Y | Negative |
|  | Loss of staff motivation due to due to unavailability of supplies, drugs, equipment, poor remuneration | Lanre-Abass (2008); Turkson (2009) | FHW Interviews | Y | Negative |
|  | Insufficient training, skills improvement for care of newborn in the lower facilities | MOH(2008); MNCH (2006); Sharma, 2012; Dzadeyson, 2007 | FHW Interviews | Y | Positive |
|  | Remuneration and level of safety of health workers | Lanre-Abass (2008) | FHW Interviews |  | Positive |
|  | Resource allocation and planning | Lanre-Abass (2008) | FHW Interviews | Y | Positive |
|  | Inadequate and untimely referrals – (insufficient ambulances) | Turkson (2009) | FHW Interviews | Y | Negative |
|  | Unequal distribution of health resources between the regions (rural and urban). | Baral *et al.* (2012) | FHW Interviews | Y | Negative |
|  | Lack of focus on the newborn care within maternity services. |  | FHW Interviews | Y | Negative |
|  | Safety methods in institutional births | Marsh *et al*. (2002), Spector *et al.* (2012) | FHW Interviews | Y | Positive |
|  | Unhygienic delivery practices | Nakakeeto (2006); Sharma (2012); Tinker *et al.,* (2009); Dzadeyson (2007); Spector *et al.,* (2012) MOH(2008); MNCH (2006) | FHW Interviews  VHW  Interviews | Y | Negative |
