# Supplementary material for: Advancing the application of systems thinking in health: understanding the dynamics of neonatal mortality in Uganda
Source: Health Res Policy Syst. 2014 Aug 8;12:36. doi: 10.1186/1478-4505-12-36 (PMC4134459; doi:10.1186/1478-4505-12-36)
Supplement: Additional file 4 — Causal loop diagram validation instrument. [file 1478-4505-12-36-S4.docx]

Additional file 1; Causal loop Diagram validation instrument

***Preamble*** *: This instrument is designed to validate the neonatal healthcare causal loop diagram. The causal loop diagram presents the dynamics associated with the provision of neonatal and maternal healthcare service. The causal loop diagrams have been presented as demand and supply models. The causal loop diagram can be used to determine short and long term leverages that could possibly lower the neonatal mortality rates and improve neonatal healthcare. The validated causal loop diagram will be used to construct a simulation model which requires extensive detail in the model building.*

***Objectives of the instrument : The instrument will be used to:***

1. Test for clarity : the extent to which the model clearly captures and communicates issues associated with neonatal healthcare.
2. Test the existence of the variables that are shown in the diagram.
3. Test the whether the relationships between variables in the model have been clearly represented.

***Target audience***

- Neonatal and maternal health experts
- National health service policy makers

***Guidelines***

Relationships between variables are denoted using links (arrows).

- A positive link (+) means that if the cause increases, the effect increases above with the same magnitude what it would otherwise have been, and if the cause decreases, the effect decreases below what it would have otherwise been.
- A negative link (-) means that if the cause increases, the effect decreases below with the same magnitude what it would otherwise have been, and if the cause decreases, the effect increases above what it would have otherwise been.

**Causal Loop Models for the Neonatal Healthcare Service**

**Figure 1: Demand - Neonatal Healthcare Figure 2: Supply - Neonatal Healthcare**

**
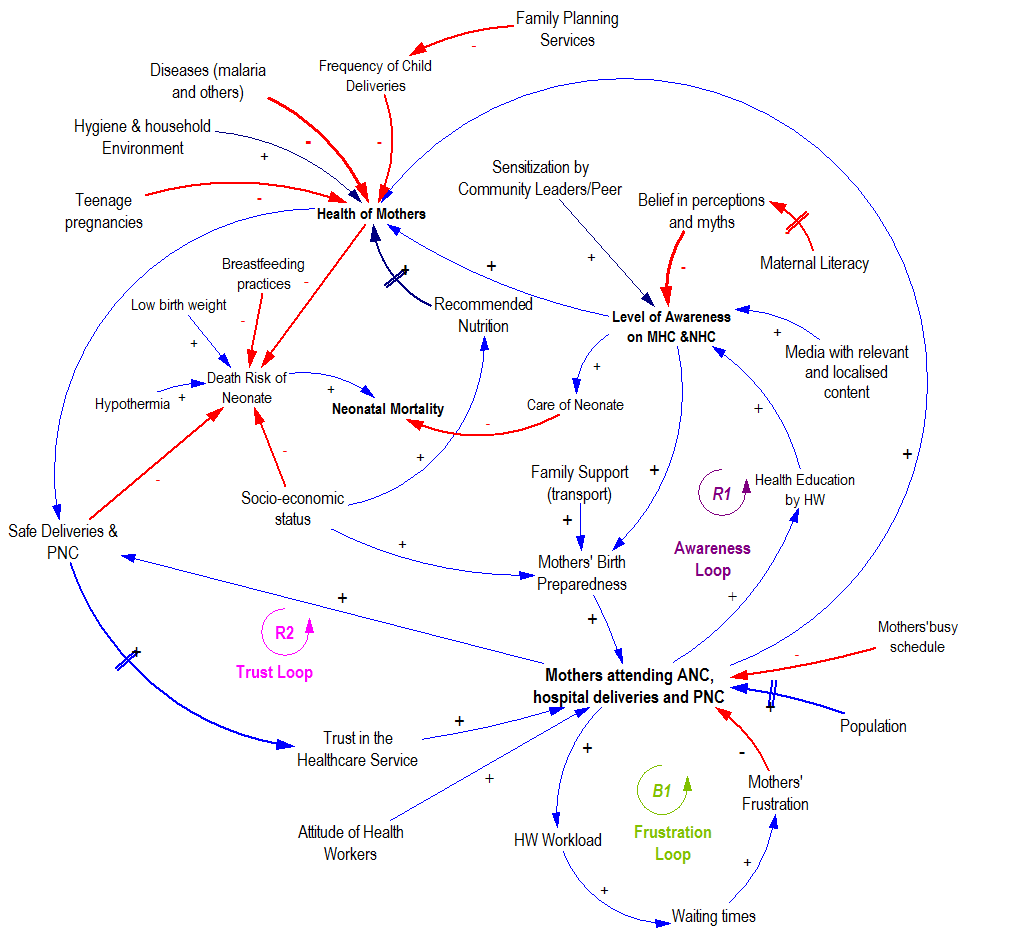

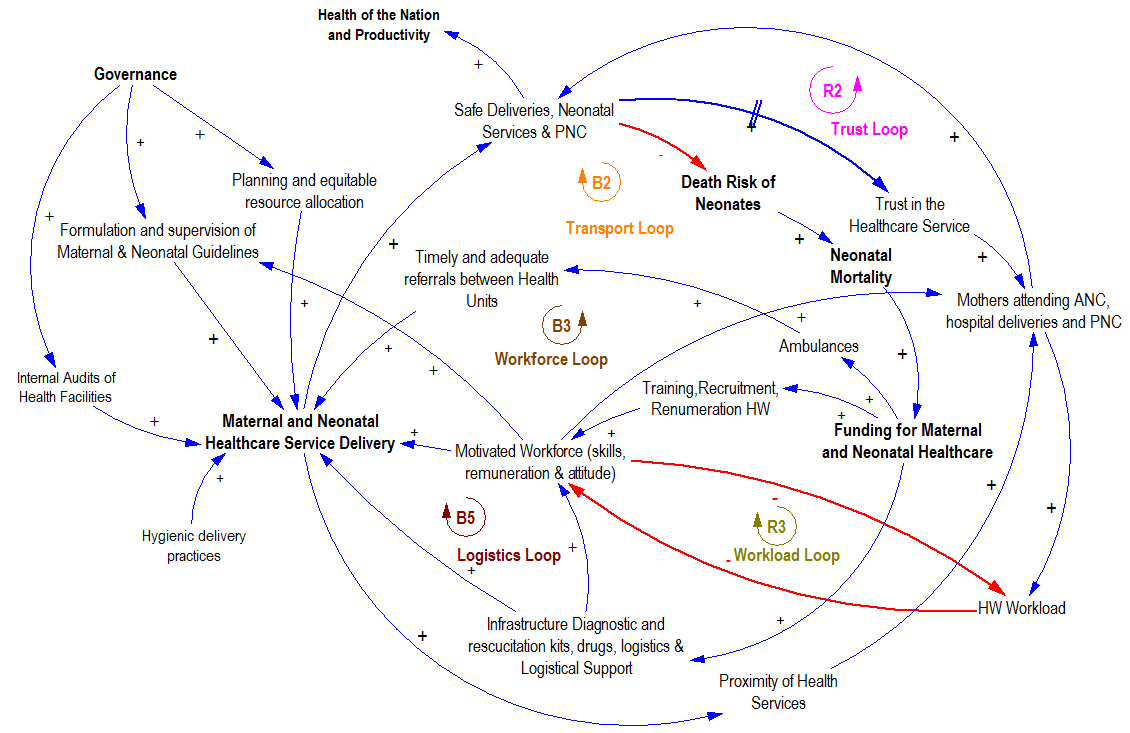
**

| **No** | **Causal Link** | **Verbal Statement** | **Questions** | **Response** |
| --- | --- | --- | --- | --- |
| 1 | 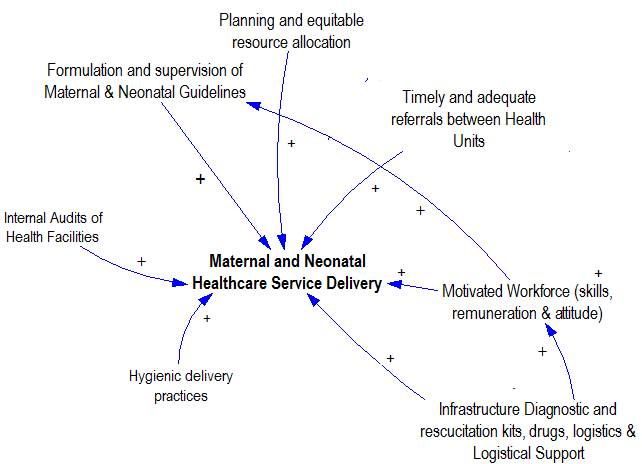 | Quality maternal and neonatal healthcare service delivery can be increased if the following are increased :-   - Motivated workforce with skills, good remuneration and attitude and balanced workload. - Infrastructural and logistical support - Formulation and supervision of maternal and neonatal guidelines - Planning and equitable resource allocation - Timely and adequate referrals between health units - Internal audits of health facilities - Hygienic delivery and practices | Do all the variables stated in this diagram exist ? |  |
|  |  |  | Do the relationships between these variables exist ? |  |
|  |  |  | Are there any significant cause factors missing ? If so, list them. |  |
|  |  |  | Are the directions of the links right or they need to be reversed (implying that the effect is the cause). |  |
|  |  |  | What other effects could be observed as a result of this cause? |  |
| 2 | 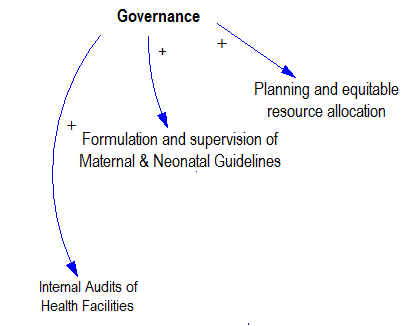 | An increase in governance results in   - Increased formulation, supervision of maternal and neonatal guidelines. - Increased planning and equitable resource allocation - Increased internal audits of the health facilities for quality maintenance | Do all the variables stated in this diagram exist? |  |
|  |  |  | Do the relationships between these variables exist? |  |
|  |  |  | Are there any significant cause factors missing? If so, list them. |  |
|  |  |  | Are the directions of the links right or they need to be reversed (implying that the effect is the cause). |  |
|  |  |  | What other effects could be observed as a result of this cause? |  |
| **3** | 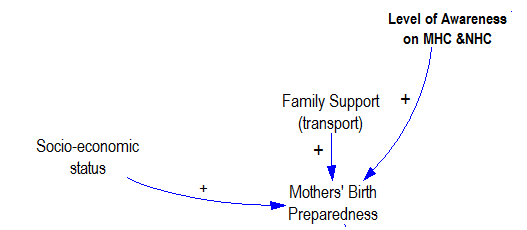 | Mothers need to be prepared before accepting to have healthcare deliveries. Birth preparedness can be increased through the following:   - Increased level of awareness on MHC and NHC services - Increased family support such as provision of transport - Increased social economic status which enables the mothers to buy materials necessary for delivery (macintosh, razors, pads etc.). | Do all the variables stated in this diagram exist? |  |
|  |  |  | Do the relationships between these variables exist? |  |
|  |  |  | Are there any significant cause factors missing? If so, list them. |  |
|  |  |  | Are the directions of the links right or they need to be reversed (implying that the effect is the cause). |  |
|  |  |  | What other effects could be observed as a result of this cause? |  |
| 4 | 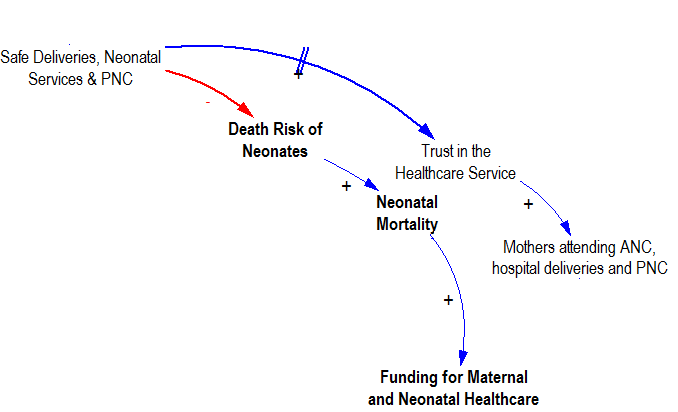 | - Increased safe deliveries increase the mothers’ trust in healthcare services which further increases their willingness to attend ANC, hospital deliveries and PNC. - Increased safe deliveries improve neonatal health thereby lowering the death risk of neonates thus lowering neonatal mortality rates. An increase in the neonatal mortality results in an increase in funding by the government and other stakeholders. | Do all the variables stated in this diagram exist? |  |
|  |  |  | Do the relationships between these variables exist? |  |
|  |  |  | Are there any significant cause factors missing? If so, list them. |  |
|  |  |  | Are the directions of the links right or they need to be reversed (implying that the effect is the cause). |  |
|  |  |  | What other effects could be observed as a result of this cause? |  |
| 5 | 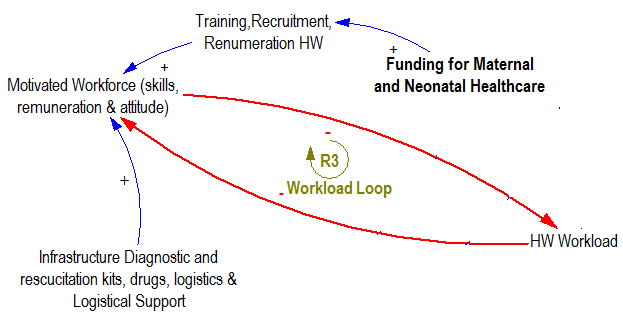 | An increase in the funding for maternal healthcare results in increased :-   - training of health workers, - recruitment of trained health workers - remuneration of health workers   As a results there is an increase in the motivation of health workers.  On the other hand an increase in the workload of health workers leads to lack of motivation of health workers leading to high attrition rates thus lowering the number of the motivated workforce. Increasing the number of the motivated workforce lowers the health worker to patient ratio thereby reducing the workload. Also reduced workload as well as an increase in the infrastructure, diagnostic kits, drugs, logistics and logistical support results in a motivated workforce. | Do all the variables stated in this diagram exist? |  |
|  |  |  | Do the relationships between these variables exist? |  |
|  |  |  | Are there any significant cause factors missing? If so, list them. |  |
|  |  |  | Are the directions of the links right or they need to be reversed (implying that the effect is the cause). |  |
|  |  |  | What other effects could be observed as a result of this cause? |  |
| 6 | 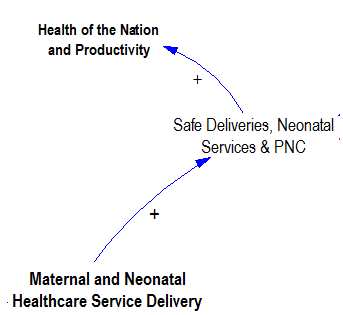 | An increase maternal and neonatal healthcare service delivery results in an increase in the number of safe deliveries and neonatal services thereby increasing the health and productivity of the nation. | Do all the variables stated in this diagram exist? |  |
|  |  |  | Do the relationships between these variables exist? |  |
|  |  |  | Are there any significant cause factors missing? If so, list them. |  |
|  |  |  | Are the directions of the links right or they need to be reversed (implying that the effect is the cause). |  |
|  |  |  | What other effects could be observed as a result of this cause? |  |
| 7 | 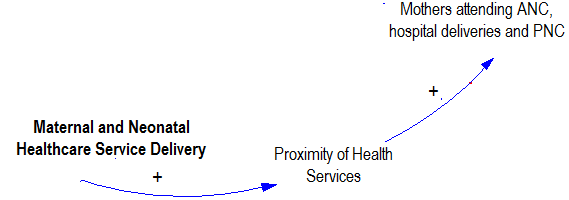 | An increase in quality maternal and neonatal service delivery through construction of more health facilities and possibly mobile healthcare services would increase the proximity to health facilities. On the other hand increased proximity to health services results in improved health facility attendance to ANC, deliveries and PNC. | Do all the variables stated in this diagram exist ? |  |
|  |  |  | Do the relationships between these variables exist ? |  |
|  |  |  | Are there any significant cause factors missing ? If so, list them. |  |
|  |  |  | Are the directions of the links right or they need to be reversed (implying that the effect is the cause). |  |
|  |  |  | What other effects could be observed as a result of this cause ? |  |
| 8 | 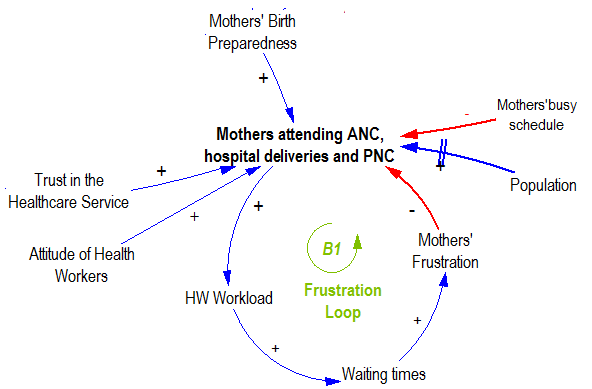 | Factors contributing to the increase in mothers’ attending ANC, hospital deliveries and PNC are   - increased trust in the healthcare service - increased attitude of health workers - increased mothers’ birth preparedness resulting from awareness, family support (transport) and socio-economic reasons - increased population   On the other hand frustration resulting from long waiting times due to high patient to health worker ratios (workload) discourages mothers from attending healthcare service. | Do all the variables stated in this diagram exist ? |  |
|  |  |  | Do the relationships between these variables exist ? |  |
|  |  |  | Are there any significant cause factors missing ? If so, list them. |  |
|  |  |  | Are the directions of the links right or they need to be reversed (implying that the effect is the cause). |  |
|  |  |  | What other effects could be observed as a result of this cause ? |  |

| 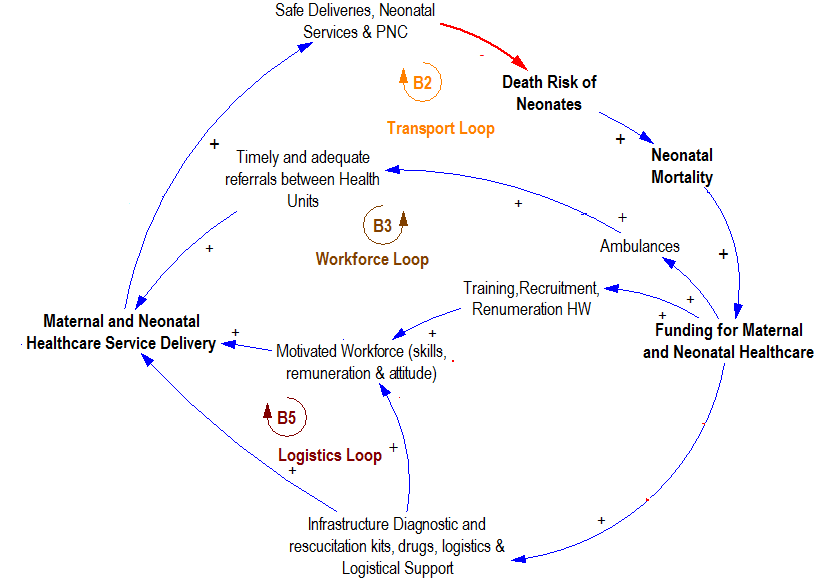 | **9. Verbal Statement** |
| --- | --- |
|  | An increase in the funding for maternal and neonatal healthcare results in an increase in the resources thereby increasing the maternal and neonatal service delivery which results in increased safe deliveries lowering the death risk of neonates. Increasing the death risk of neonates increases neonatal mortality which necessitates more funding for more resources The resources are explained below:   - Transport loop: Increased provision of ambulances increases the timely and adequate referrals between hospitals - Workforce loop: Increased training, recruitment and remuneration results increased motivated workforce - Logistics loop: Increased infrastructure diagnostic and resuscitation kits, drugs and logistics. |

| **Questions** | **Response** | |
| --- | --- | --- |
| Do all the variables stated in this diagram exist ? |  | |
| Do the relationships between these variables exist ? |  | |
| Are the directions of the links right or they need to be reversed (implying that the effect is the cause). |  |  |
| What other effects could be observed as a result of this cause ? |  |  |
| Are there any significant cause factors missing ? If so, list them. |  | |

| 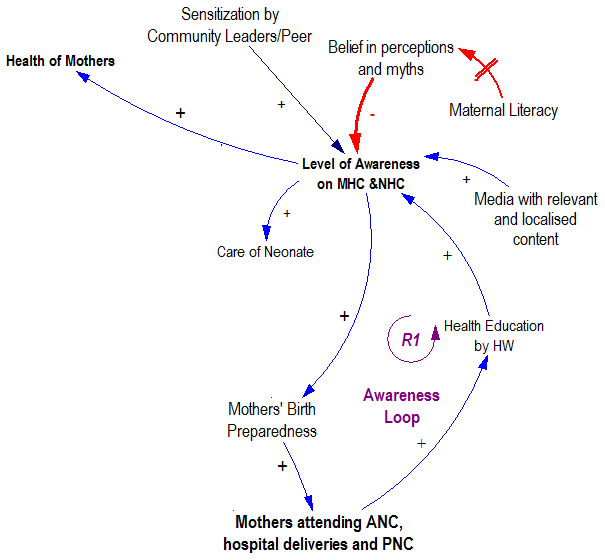 | **10. Verbal Statement** |
| --- | --- |
|  | The level of awareness on maternal and neonatal matters increases through the following means   - Through health education sessions during attendance of ANC, hospital deliveries and PNC. - Through sensitization by community leaders as well as peer to peer interactions with fellow mothers - Through the media adverts provided by the Non-Government Organisations involved in healthcare and Ministry of health - Belief in perceptions and myths due to high illiteracy levels result in a decrease in level of awareness.   An increase in level of awareness on maternal and neonatal healthcare results in increased health of mothers and mothers’ birth preparedness.  The figure also presents an awareness loop which presents a growing action that is reinforcing. The more awareness one acquires through health education and other means, the more likely they are to use health services which in turn increases the level of awareness. |

| **Questions** | **Response** |
| --- | --- |
| Do all the variables stated in this diagram exist ? |  |
| Do the relationships between these variables exist ? |  |
| Are there any significant cause factors missing ? If so, list them. |  |
| Are the directions of the links right or they need to be reversed (implying that the effect is the cause). |  |
| What other effects could be observed as a result of this cause ? |  |

| 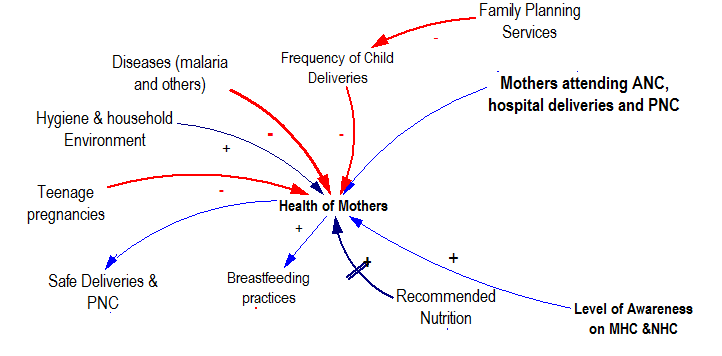 | **11. Verbal Statement**  The health of the mothers can be improved through   - Attendance of ANC, hospital deliveries and PNC - Right maternal age (not teenage pregnancies) - Living in a healthy and hygienic environment - Use of family planning services for improved child spacing - Having the recommended nutrition which can be made available with improved Socio-economic status. - Increased maternal and neonatal awareness   On the other hand, untreated diseases, frequent deliveries and teenage pregnancies lower the health of the mothers.  With improved health of the mothers, they are able to have safe deliveries and post natal care as well as breastfeeding practices which contribute greatly to the care and health of the neonates |
| --- | --- |

| **Questions** | **Response** |
| --- | --- |
| Do all the variables stated in this diagram exist ? |  |
| Do the relationships between these variables exist ? |  |
| Are there any significant cause factors missing ? If so, list them. |  |
| Are the directions of the links right or they need to be reversed (implying that the effect is the cause). |  |
| What other effects could be observed as a result of this cause ? |  |

**Evaluation of the Conceptual Model**

In your opinion, how do you rate this model ?

1. Reasonable (realistic) : [a] Very reasonable

[b] Reasonable

[c] Fairly reasonable

[d] not reasonable

2. Representation of the issues in neonatal healthcare service [a] Very good

[b] Good

[c] Fairly good

[d] not at all good

3. Communication tool concerning neonatal healthcare issues [a] Very useful

[b] Useful

[c] Fairly useful

[d] not at all useful

4. Aid tool that can be used by stakeholders in decision making [a] Very useful

[b] Useful

[c] Fairly useful

[d] not at all useful

*Thank you*
